# Alpha-synuclein-induced stress sensitivity renders the Parkinson’s disease brain susceptible to neurodegeneration

**DOI:** 10.1186/s40478-024-01797-w

**Published:** 2024-06-17

**Authors:** Modestos Nakos Bimpos, Katerina Karali, Christine Antoniou, Dionysios Palermos, Maria Fouka, Anastasios Delis, Iason Tzieras, George Panagiotis Chrousos, Yassemi Koutmani, Leonidas Stefanis, Alexia Polissidis

**Affiliations:** 1 Center of Clinical, Experimental Surgery and Translational Research, Biomedical Research Foundation of the Academy of Athens - BRFAA, 11527 Athens, Greece; 2 German Center for Neurodegenerative Diseases, Feodor-Lynen-Straße 17, 81377 Munich, Germany; 3 Athens International Master’s Programme in Neurosciences, Department of Biology, National and Kapodistrian University of Athens, 15784 Illisia, Athens, Greece; 4 University Research Institute on Maternal and Child Health and Precision Medicine, and UNESCO Chair on Adolescent Health Care, Aghia Sophia Children’s Hospital, National and Kapodistrian University of Athens, 11527 Athens, Greece; 5 1St Department of Neurology, Eginition Hospital, Medical School, National and Kapodistrian University of Athens, 11527 Athens, Greece; 6 Department of Science and Mathematics, ACG-Research Center, Deree – American College of Greece, 15342 Athens, Greece

**Keywords:** Parkinson’s disease, Alpha-synuclein, Corticosterone, HPA axis, Glucocorticoids, Chronic stress

## Abstract

A link between chronic stress and Parkinson’s disease (PD) pathogenesis is emerging. Ample evidence demonstrates that the presynaptic neuronal protein alpha-synuclein (asyn) is closely tied to PD pathogenesis. However, it is not known whether stress system dysfunction is present in PD, if asyn is involved, and if, together, they contribute to neurodegeneration. To address these questions, we assess stress axis function in transgenic rats overexpressing full-length wildtype human asyn (asyn BAC rats) and perform multi-level stress and PD phenotyping following chronic corticosterone administration. Stress signaling, namely corticotropin-releasing factor, glucocorticoid and mineralocorticoid receptor gene expression, is also examined in post-mortem PD patient brains. Overexpression of human wildtype asyn leads to HPA axis dysregulation in rats, while chronic corticosterone administration significantly aggravates nigrostriatal degeneration, serine129 phosphorylated asyn (pS129) expression and neuroinflammation, leading to phenoconversion from a prodromal to an overt motor PD phenotype. Interestingly, chronic corticosterone in asyn BAC rats induces a robust, twofold increase in pS129 expression in the hypothalamus, the master regulator of the stress response, while the hippocampus, both a regulator and a target of the stress response, also demonstrates elevated pS129 asyn levels and altered markers of stress signalling. Finally, defective hippocampal stress signalling is mirrored in human PD brains and correlates with asyn expression levels. Taken together, our results link brain stress system dysregulation with asyn and provide evidence that elevated circulating glucocorticoids can contribute to asyn-induced neurodegeneration, ultimately triggering phenoconversion from prodromal to overt PD.

**Supplementary Information:**

The online version contains supplementary material available at 10.1186/s40478-024-01797-w.

## Introduction

On an aging planet, where neurological disorders have become the major cause of disability [1], Parkinson’s disease (PD) is considered the fastest growing neurodegenerative disease [2]. Although more than 95% of PD cases have no identifiable genetic cause [3], the neuronal protein alpha-synuclein (asyn) appears to be a major player in both genetic and idiopathic PD pathogenesis [4]. As it has been quite challenging to recapitulate full-blown PD in rodent models of asyn overexpression, it is believed that additional environmental triggers may be needed for asyn to elicit its pathogenic effect [5].

In 1886, the famous neurologist Sir William Gowers described in the “Manual of Diseases of the Nervous System” that “prolonged anxiety and severe emotional shock often precede the onset of tremor”, one of the most characteristic motor symptoms of PD (then referred to as “paralysis agitans”) [6]. Today, we know that chronic stress and stress hormones affect central nervous system function at all levels: transcriptional regulation, cellular signaling, synaptic function, neurotransmission, glial function, and behavior [7]. While chronic stress has firmly established a causal relation with the pathogenesis of neuropsychiatric diseases such as depression [8], it may also cause dopaminergic neurodegeneration, the hallmark pathology of PD, in susceptible individuals [9], and may accelerate dopaminergic neurodegeneration in PD animal models [10, 11].

To date, no study has comprehensively addressed the risk chronic stress confers, and whether it should be considered a trigger, a facilitator or an aggravator of PD [5]. Clinical evidence alludes to an abnormal stress response, i.e. hypothalamic–pituitary–adrenal (HPA) axis dysregulation, however, direct evidence of molecular and glucocorticoid-mediated pathologies is lacking [12]. Asyn poses a likely culprit as exacerbated asyn pathology has been demonstrated in the A53T mouse model following chronic mild stress [11] and in a model of seeded asyn pathology in mouse striatum following corticosterone administration [13]. However, it is currently unknown if enhanced asyn burden itself is involved in stress dysregulation and vice versa.

To address this issue, we assessed HPA axis function in bacterial artificial chromosome transgenic rats that overexpress human full-length wildtype asyn (BAC). Nine month-old BAC rats model a premotor hyperdopaminergic phenotype, representing a state prior to overt neurodegenerative phenoconversion [14]. Furthermore, we examined the pathological and phenotypic consequences of chronic exogenous corticosterone (CORT) administration to demonstrate that chronic stress can act additively or synergistically with asyn to incite changes related to asyn pathology, neuroinflammation, nigrostriatal neurodegeneration, and non-motor and motor behavioral deficits. Finally, upon finding molecular perturbations in hippocampal stress signaling in BAC rats, we sought to investigate if such changes are conserved in PD patient brains and could potentially be involved in altered sensitivity to chronic stress in the context of PD.

## Materials and methods

### Human brain specimens

A total of 12 cases (n = 6 healthy controls, n = 6 PD cases) were included for the present study. Postmortem autopsy material encompassing the hippocampus was collected with full ethical permission, following donation by next of kin, and were kindly provided by the Parkinson’s Disease UK Brain Bank (PDUKPD: Division of Brain Sciences, Imperial College London Hammersmith Campus, Du Cane Road, London W12 0NN). The demographic details of these cohorts are available in Table S1 [15]. The brain tissues arrived on dry ice and were stored at − 80 °C, until further analysis. Tissue handling/experimentation was approved by the Bioethics Committee of the Biomedical Research Foundation of the Academy of Athens.

### Animals and ethics approval

All experiments were approved by the Ethical Committee for Use of Laboratory Animals at the BRFAA (Permit no. 1043/20.02.2019) and complied to the ARRIVE guidelines. Middle-aged (9-month-old) male wild type and BAC Sprague–Dawley rats were housed in pairs in individually ventilated cages, on a 12-h light/dark cycle (lights on at 7:00 am), at a constant room temperature of 21 ± 1 °C and had unlimited access to food and water. Upon computation of appropriate sample sizes, animals were randomly assigned to the experimental groups. The interaction between sex, environment and genetics is complex and understudied, thus, for this first study of ours exploring the interaction between genetics and stress, male animals were chosen due to higher overall incidence and prevalence of PD, earlier disease onset, worse motor symptoms and progression, and more frequent cognitive decline in males [16]. We based this decision on ours and others animal studies that revealed a significant PD phenotype only in male rats [14] and chronic stress PD exacerbation only in male A53T mice [11]. The animals received 14-d CORT in drinking water, followed by behavioral tests and then killed either by decapitation, for neurochemical and biochemical analysis, or by perfusion, for immunohistochemical analysis 24 h later.

### Chronic corticosterone administration

CORT (Corticosterone, 27840, Sigma Aldrich) was initially dissolved in absolute ethanol and then added at a concentration of 50 μg/ml to the drinking water (1%). The animals were designated to one of four treatment groups: (1) wildtype control (WT CTL), (2) wildtype CORT (WT CORT), (3) BAC control (BAC CTL) and BAC CORT (BAC CORT) groups. Either CORT or a vehicle solution (drinking water with 1% ethanol) was administered to the CORT and the CTL groups, respectively, for two weeks [17]. Fresh solutions were prepared every two days in tinfoil-covered water bottles to protect CORT from light-induced degradation. There were no differences in daily water consumption among groups. Average water intake of 35 ml/rat/day corresponded to a daily dose of approximately 1.75 mg of CORT per animal.

### Behavioral tests

All behavioral testing was carried out during the light phase, between 9:00 and 17:00. Two different cohorts of animals (3–5 littermates/group per cohort) were tested in a behavioral test battery of motor and non-motor tests, in sequential order: open field, elevated plus maze, prepulse inhibition, gait analysis and postural instability. Each behavioral task was separated by at least 24 h.

#### Open field

The open field test was employed to evaluate locomotion and anxiety-related behaviors in a square acrylic open field arena (40 × 40 × 30 cm) over a one-hour period, according to previously established protocols [14]. The parameters evaluated were locomotor activity, as measured by the total distance travelled (cm), exploration, the rearing frequency (when the animal stands up on its hind legs) and anxiety-related behavior, as measured by the total amount of time the rat spent in the central area of the arena (s).

#### Elevated plus maze (EPM)

Anxiety-related behaviors were assessed in a polyvinyl chloride EPM apparatus over a five-minute period, according to previously established protocols [14]. The parameters evaluated were the percentage of time spent in the open arms (vs. total time in closed and open arms) and the locomotor activity (measured as the total distance travelled, cm).

#### Prepulse inhibition (PPI)

PPI was conducted according to previously established protocols [14]. Briefly, on the first day, during the habituation phase, animals were exposed to 5 min of 70-dB white noise, followed by a series of sound pulses of varying amplitude (70, 80, 90, 100, 110 and 120 dB, in pseudorandom order, 1 s apart; 20 s intertrial interval; five times each). The average startle response of the five trials, termed acoustic startle response (ASR), was calculated. The next day, the animals were once more restrained, placed in the startle chamber and introduced to a PPI protocol consisting of 5 min habituation (background white noise 70 dB), 10 pulse‐alone (115 dB) trials, 10 prepulse (each of 75, 80, 85 and 90 dB) plus pulse trials in pseudorandom order (1 s apart; 20 s intertrial interval) and 10 no stimulus (white noise) trials. PPI was calculated as a percentage score: % PPI = [1 − (PreS)/S)] * 100, where “PreS” denotes the mean startle response for prepulse plus trials and “S” the mean startle response for the startle pulse-alone trials.

#### Gait analysis

Gait analysis was assessed with footprinting. Each rat had its forelimbs and hindlimbs coated with grey and black non-toxic tempera paint, respectively, and then allowed to walk along a paper strip placed in a 13 cm wide PVC corridor (with 5 cm high walls). A series of at least four sequential steps were used (not including the first or last strides) for manual measurement of stride length, width between forelimbs and hindlimbs and the overlap between forelimb and hindlimb for each stride (cm).

#### Postural instability

To assess postural instability, after applying paint to the forelimbs, each rat was held upside-down from its tail, almost vertically, over a paper strip. The rat was then slowly lowered until its paws touched the paper and began to move forward, making steps while trying to regain its center of gravity, in response to the sequential weight shifts from one forelimb to the other. A series of at least three sequential steps were used to measure the distance it takes for the animal to take a step with the forelimb to regain its balance. To avoid baseline variation between WT and BAC animals, since the transgenic animals weigh less and thus, have a smaller body size [14], we calculated the overall variation among the forelimb steps the rats took trying to regain their balance rather than the length of the forelimb step.

### Animal brain tissue harvesting

Brains were harvested the day after completing the battery of behavioral tests either by decapitation or by perfusion. Decapitated brains were dissected on ice and the following regions were collected: hypothalamus, midbrain (containing the substantia nigra pars compacta (SNpc)), striatum and hippocampus. All the regions were weighed and snap frozen in liquid nitrogen before being stored in -80 °C.

### Corticotropin releasing factor (CRF) measurements in rat hypothalami

Rat hypothalami were homogenized, diluted and analyzed to measure CRF levels using the CRF (Human, Rat, Mouse, Canine, Feline) EIE Kit (Phoenix Pharmaceuticals, Inc., Catalog No. EK-019–06), following the manufacturer’s instructions. The enzyme-linked immunosorbent assay (ELISA) was quantified with a SPARK® multimode microplate reader (TECAN).

### Corticosterone measurements in rat plasma

Trunk blood was collected from the animals upon sacrifice in EDTA coated 1.5 ml tubes. Animals from each group were sequentially sacrificed during morning hours (09:00–11:00) to eliminate the time factor since CORT secretion follows a diurnal rhythm. The blood was centrifuged for 10 min in 2000×*g* in a precooled centrifuge set at 4 °C. The supernatant was collected, diluted and analyzed using a commercial CORT ELISA Kit (Enzo, Catalog No. ADI-900-097), according to the manufacturer’s instructions. The ELISA was quantified with a SPARK® multimode microplate reader (TECAN).

### Determination of rat brain region-specific monoamine neurotransmitter levels

Homogenization and sample analysis was performed according to established protocols [14]. The assay measured the concentration of noradrenaline (NA), dopamine (DA) and its metabolites (3, 4-Dihydroxyphenylacetic acid (DOPAC), 3-Methoxytyramine (3-MT) and homovanillic acid (HVA)) in brain tissue homogenates with reverse-phase ion-pair chromatography on an isocratic pump (YL9112 Instrument Co., Ltd., Gyeonggi-do, Korea) coupled with an electrochemical detector (BASi LC-EC, Bioanalytical Systems, Inc., Indianapolis, IN, USA). HPLC software (DataApex, Clarity) was used to quantify neurotransmitter levels by comparing the area under the peaks with the area of external reference standards. Final results are expressed as ng/mg of wet tissue weight.

### Asyn pathology assessment in the rat brain

#### Protein extraction protocol, immunoblotting and analysis

Briefly, following brain tissue harvesting by decapitation, hippocampi and striata were homogenized, lysed, centrifuged (120 000×*g* for 60 min) and depending on the lysis buffer (either STET (50 mM Tris [Sigma-Aldrich, T1503], pH 7.4, 150 mM NaCl [Lach-Ner, 30093], 1% Triton-X-100 [Applichem, A1388], 2 mM EDTA [Applichem, A1104], protease and phosphatase inhibitors [Roche, 11836153001, 4906845001]) or RIPA (same as STET supplemented with 1% SDS)), appropriate amounts of the supernatants containing either the “soluble” (Tx100) or the membrane-associated and aggregated “insoluble” (SDS) protein fractions were loaded on 12% SDS polyacrylamide gels. The proteins were transferred onto nitrocellulose membranes and blocked using 5% milk in 1 × TBST. For detection of pS129 asyn, membranes were incubated overnight in PBS 1 × at 65 °C and blocked in 5% BSA. For a list of all primary and secondary antibodies used for all immunoblot/staining protocols, refer to Table S2. Analysis was performed with Fiji/ImageJ [18, 19].

#### PS129 Asyn Immunohistochemistry

Perfusion, brain fixation, sectioning, and immunohistochemistry were performed according to standardized lab protocols [14, 20]. Briefly, the brains were sliced coronally (thickness 35 μm) on a cryotome [LEICA CM3050 S] and three representative sections from each brain region of interest were chosen per subject. The sections were placed for 10 min in 3% H_2_O_2_/10% methanol mixture and subsequently blocked with 5% normal goat serum for 1 h at room temperature (RT). The rabbit monoclonal anti-α-synuclein phospho S129 antibody was used as the primary antibody, for 48 h at 4 °C, the sections were washed three times for five min in PBS 1 × and incubated for 1 h with biotinylated goat anti-rabbit IgG secondary antibody [Vectastain ABC kit, PK-4001, Vector Laboratories]. Three washes in PBS 1 × for 5 min followed, and the sections were then treated for 1 h with ABC solution [Vectastain ABC kit, PK-4001, Vector Laboratories] at RT followed by three more washes in PBS 1x. Antibody binding was visualized by the reaction of hydrogen peroxidase with 3, 3-diaminobenzidine [DAB, Dako, K3468]. Finally, the sections were mounted on Superfrost microscope slides [Epredia, J1800 AMNZ] dehydrated and treated for 15 min with 0.5% Cresyl Violet Acetate. Following staining, the sections were rehydrated and treated with xylene. Finally, the sections were placed on slides with DPX Coverquick 2000 [VWR chemicals, 0554730-500ML] as a mounting medium.

#### Microscopic observation

Immunohistochemistry staining was observed with a Leica DM RA2 microscope. Whole sections were scanned using Stereo Investigator software [MBF Bioscience, Version 10] using a vertiga 2000-Qimaging camera and a 10 × objective lens. For the analysis, images from the “5th edition of The Rat Brain in Stereotaxic Coordinates” [21] were overlaid with the tile scanned images of the section (three representative coronal sections per brain region with the coordinates “bregma/interaural line”; for hypothalamus: − 1.40 mm/7.60 mm, − 3.14 mm/5.86 mm and − 4.16 mm/4.84 mm and for hippocampus: − 2.56 mm/6.44 mm, − 3.14 mm/5.86 mm and − 3.80 mm/5.20 mm). Using Fiji/ImageJ, the specific regions of the hypothalamus and the hippocampus were selected. Background subtraction of the area of the pS129 was quantified as compared to the total area of the region.

#### Confocal microscopy and analysis

A Leica TCS SP5 II on a DM 6000 CFS upright confocal microscope was used to acquire z stacks of 1024 × 1024 pixel resolution throughout the whole thickness of the midbrain sections with a 40 × 1.20 objective lens and a z step size of 0.7 microns. For each brain section, three representative images were taken to cover the whole region of the SNpc and image analysis and evaluation of the volume of the pS129 asyn was performed with Fiji/ImageJ. Briefly, the TH channel was used to create a mask of the SNpc, after applying a threshold. The total volume of the SNpc was measured in the z-projection (using the “Sum slices” option) and measured by dividing the Raw Integrated Density by 255. The pS129 channel was masked using the “Image calculator” option in the “Process” tab of Fiji/ImageJ (“Min” operation). After applying a threshold, the masked image was z-projected using the “Sum slices” option and the volume was calculated. The final values are expressed as the ratio of pS129volume/total TH volume. For the assessment of asyn pathology in dopaminergic neurons of the hypothalamus (Fig. S1), z-stacks of 2048 × 2048 pixel resolution and a step-size 0.3 microns were generated using a water-immersion Leica HCX APO 63 × 1.20 objective on a Leica TCS SP5 confocal microscope (Wetzlar, Germany). Analysis was performed using Imaris × 64 9.1 software. To create the surface of dopaminergic cells, Imaris Surface module was utilized, and an intensity threshold was applied to distinguish between background and cell fill. The surface that was created from the TH signal was used as a mask for pS129 asyn signal and the overlap between the reconstructed surface and pS129 immunoreactivity signal created a new signal channel representing the pS129 asyn located inside the dopaminergic neurons.

### Immunohistochemistry of the nigrostriatal pathway in the rat brain

#### DAB staining

Representative sections (every 6th section) of the entire striatum (STR) and midbrain (containing the SNpc) were stained with the polyclonal anti-tyrosine hydroxylase (TH) antibody. The staining was visualized using DAB and the sections were further stained with cresyl violet (Nissl stain) and dehydrated in consecutive increasing concentrations of ethanol solution.

#### Dopaminergic cell counts in the SNpc

Stereological counts of TH + neurons in the SNpc was estimated using a stereological microscope [LEICA DMRA2] coupled with a QImaging color camera and Stereo Investigator v10.0 software [MBF Bioscience, USA], according to established protocols [14]. For each animal, 8–10 representative sections throughout the rostro-caudal axis of the SNpc were quantified based on an unbiased method using the Optical Fractionator, employing a systematic random sampling of counting frames. Counting contours in each section were outlined with a 2.5 × objective lens while counting was performed using a 63 × 1.30 glycerol immersion objective lens with the following settings: optical dissector, grid size (150 μm) and counting frame (50 μm). A coefficient of error (Gundersen, m = 1) of ≤ 0.1 was accepted.

#### Dopaminergic afferent densitometry in the striatum

The density of TH + axons in the striatum was measured, as previously described [20]. Six sections for each animal, covering the whole rostrocaudal axis of the striatum, were stained for TH using DAB staining. Densitometric analysis was performed using Fiji/ImageJ. The striatum was sub-divided into three regions: dorsolateral (DL), dorsomedial (DM) and ventral (V), according to the boundary between the caudate-putamen complex and the nucleus accumbens. The density of TH + neurons was measured using Fiji/ImageJ for each region.

### Neuroinflammation assessment along the rat nigrostriatal *Axis*

#### Tissue processing for immunofluorescence

Immunohistochemistry was performed in free-floating sections for the assessment of neuroinflammation along the nigrostriatal axis. Midbrain and striatal Sects. (3 sections/brain region/subject) were selected, washed in PBS 1x, and blocked in a buffer containing 5% natural-goat-serum (NGS) and 0,1% Triton X in PBS 1x, to reduce nonspecific binding of the antibodies and background signal. Incubation overnight at 4 °C with primary antibodies followed, using anti-GFAP (glial fibrillary acidic protein) for the staining of astrocytes, anti-Iba1 (ionized calcium binding adaptor molecule 1) for microglia and anti-TH for dopaminergic neurons in the SNpc. The sections were washed in PBS 1 × and incubated with secondary antibodies conjugated with fluorophores (CF488A green and CF555 red) for 1 h at RT. After PBS 1 × washes, sections were mounted on poly-L-lysine glass microscopic slides [Epredia, J2800AMNZ] with mounting medium containing DAPI (4′,6-diamidino-2-phenylindole) [Fluoromount-G™, Invitrogen].

#### Structured illumination (SIM) confocal microscopy

The Aurox Clarity Laser Free Structure Illumination Confocal Microscope was used to generate tile scan images of the stained brain slices which were subsequently quantified to measure the mean intensity of the GFAP and Iba1 glial cells using TH + staining to select the ROIs, in the SNpc and the striatum. The images were analyzed using Fiji/ImageJ. Briefly, images belonging to the same section were stitched together (using the “Stitching” plug-in), converted from 16-bit to 8-bit (after setting the contrast to its full range) and the background was subtracted (“Subtract background” option in the “Process” tab). ROIs were selected based on a max projected version of the image and saved for subsequent use in the workflow. Total volume of the structure of interest (SOI) was evaluated by thresholding the stitched image, z-projecting using the “Sum slices” option and measuring the integrated density statistic after overlaying the ROI on the z-projected image. The resulting value was equal to the total number of pixels comprising the structure of interest over the entire 3D volume × 255. Therefore, to extract the total volume (V), the latter value was divided by 255. To calculate the numerator of the mean volume ratio, the image was masked based on the thresholded image using the “Image calculator” option in the “Process” tab of Fiji/ImageJ (“Min” operation). The resulting image contains 0-valued pixels over background regions (0-value in the thresholded image) and the raw values over the structure of interest. The masked image was z-projected using the “Sum slices” option and once more the raw integrated density statistic giving the intensity sum (I) over the SOI was calculated after overlaying the ROI. The mean intensity of the SOI, within the ROI, is given by the ratio: I/V.

### RNA extraction and cDNA synthesis from human and rat hippocampi

RNA was isolated from both human and rat hippocampus samples using the Nucleospin© RNA kit [Macherey Nagel], according to manufacturer’s instructions. Total RNA was quantified with NanoPhotometer [IMPLEN]. RNA integrity was assessed by loading 200 ng of each sample on a 1% agarose gel. 1 μg of total RNA was used for cDNA synthesis in a two-step cDNA synthesis protocol, according to manufacturer’s instructions [GoScript™ Reverse Transcriptase, Promega].

### RT qPCR analysis of human and rat hippocampal gene expression

To assess changes in expression of selected genes (a list of all the primers is available in Table S3), real time (RT) qPCR was performed in triplicates for each sample. GAPDH (rat samples) and RPL13a (human samples) were used as housekeeping genes for normalization. 2 μl of each sample was added to a master mix preparation containing 2 μl of each primer, 7.5 μl of iTaq Universal SYBR Green Master Mix [Biorad, 1725121] and 3.5 μl nuclease-free water [Invitrogen], and loaded on a 96-well plate [Roche, Lightcycler 480 Multiwell plate 96]. Data was collected and analyzed using the LightCycler 96 System [Roche]. The value of the cycle number at which any sample crossed the threshold (Ct) was used to determine fold difference. In addition, the geometric mean of the reference gene served as a point for normalization. Fold difference was calculated with the 2^−ΔΔCt^ method [22].

### Statistical analysis and graphic illustrations

GraphPad Prism 9 software was used for all statistical analyses and Adobe Illustrator Artwork 23 was used to create figures and artwork. For two-way ANOVAs, the two factors were genotype (WT and BAC) and drug treatment (CTL and CORT) with Bonferroni’s multiple comparison tests used for post-hoc analysis. Unpaired Student’s t-tests and simple linear regression analysis were also used, where applicable. A *p*-value of < 0.05 was considered statistically significant. The exact *p*-values for all analyses are reported in Table S4. Results are presented in graphs as mean ± SEM.

## Results

After two weeks of CORT administration, we performed a battery of behavioral tests (OF, EPM, gait and postural instability analysis) before sacrificing the animals and collecting brain regions related to the HPA axis and affected in PD (hypothalamus, hippocampus, SNpc and striatum) for further analysis. PS129 asyn levels were measured in all of the aforementioned brain regions while in the hypothalami of the animals we measured CRF levels along with the levels of neurotransmitters related to the stress response and PD pathology (NA and DA turnover). In the rat hippocampi, we calculated NA and total asyn protein levels. Along the nigrostriatal pathway of the rats, we measured the degeneration of TH + neurons, glial activation and total asyn levels. Finally, CRF, GR and MR gene expression levels were measured in the hippocampi of both human and rat samples.

### Human asyn overexpression in BAC rats confers baseline HPA *axis* dysregulation

**Fig. 1:**
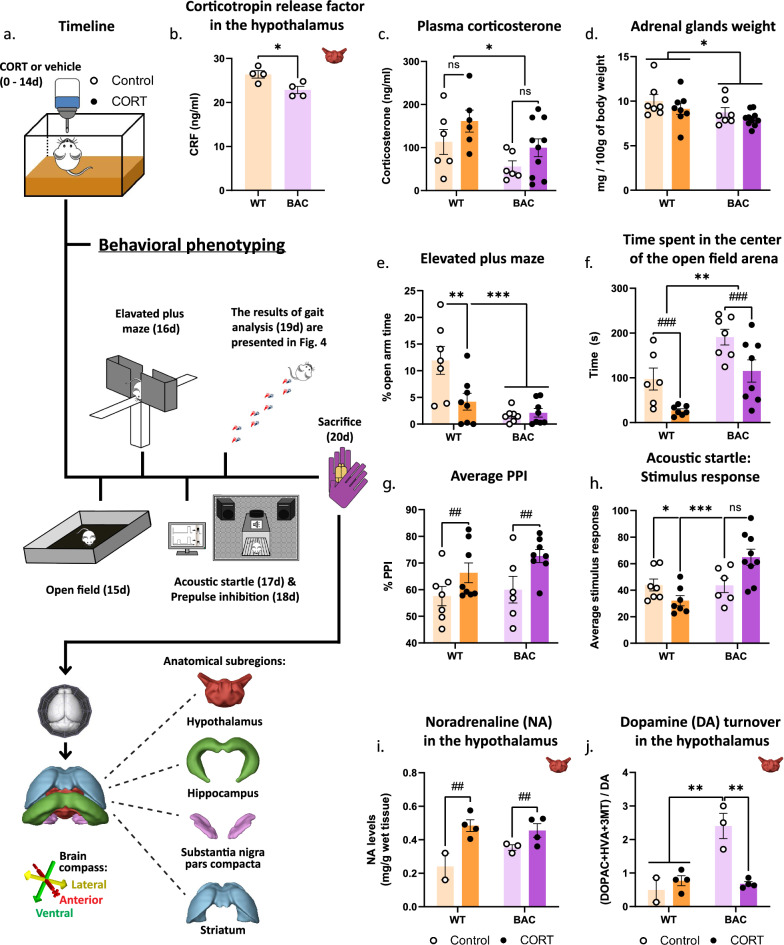
HPA axis dysregulation in asyn BAC rats. As depicted in the experiment outline **a**, following CORT administration for 14 days and a battery of behavioral tests consisting of open field, elevated plus maze (EPM), prepulse inhibition (PPI) and gait analysis, brains of BAC and WT rats were harvested and the hypothalamus, hippocampus, midbrain—enclosing the SNpc—and striatum regions were dissected. First, we evaluated **b** CRF levels (ng/ml) in the hypothalamus of 9 mo BAC animals and age-matched WT controls without treatment, **c** levels of CORT in plasma (ng/ml) following CORT administration and **d** adrenal glands weight (mg/100 g of body weight). For the behavioral phenotyping we calculated **e** the percentage of time spent in the open arms of the EPM, **f** the total amount of time (s) spent in the center of the OF arena, **g** average % PPI and **h** average stimulus response in PPI test. Following neurochemical measurements in the hypothalamus, we quantified **i** NA levels (mg/g wet tissue) and **j** DA turnover (DOPAC + HVA + 3MT/DA). Unpaired t-test was applied for **b** while two-way ANOVAs were applied for **c**–**j** with Bonferroni's multiple comparisons post-hoc tests. All data are expressed as mean ± SEM. Asterisk (*) is used to mark genotype main effects while hashtag (#) marks treatment main effects. Significance levels: */# *p* < 0.05; **/## *p* < 0.01; ***/### *p* < 0.001. N = 4 per group for b, N = 7–10 for **c**–**h** and N = 2–4 for **i** and **j**

The first evidence of HPA axis dysregulation was lower baseline CRF levels found in the hypothalamus of BAC rats (Fig. 1b). Next, HPA axis activity was assessed by measuring the levels of CORT circulating in the blood. The experimental groups that received CORT revealed nonsignificant elevated plasma CORT levels (Fig. 1c; *p* = 0.0628) and the BAC animals demonstrated decreased CORT levels, regardless of treatment (Fig. 1c). Adrenal insufficiency and relative HPA axis dysregulation was also confirmed by a significant decrease in the weight of the adrenal glands in BAC rats, regardless of treatment (Fig. 1d). Subsequently, the EPM, open field and PPI tests were used to assess behaviors affected by stress, specifically anxiety and sensorimotor gating. While BAC animals displayed a baseline increased mobility in the EPM, CORT further increased the distance travelled in both WT and BAC rats in the EPM (Fig. S2). As expected, CORT increased the levels of anxiety in WT rats, depicted by a decrease in the percentage of time that WT CORT animals spent in the open arms of the EPM, while the baseline anxiogenic profile of BAC rats was not further exacerbated (Fig. 1e). In the open field, CORT decreased the total time spent in the center of the arena, indicating elevated anxiety, while there was a baseline difference with BAC animals spending more time in the center of the open field (Fig. 1f) that correlated with the total distance travelled in the arena of the open field (Fig. S3). Assessment of sensorimotor gating revealed an increased average percentage of PPI in both WT and BAC rats following treatment with CORT (Fig. 1g). An increase in startle response amplitude following CORT treatment in BAC rats was indicative of hypervigilance (Fig. 1h). Moreover, CORT increased noradrenaline (NA) levels in the hypothalamus in both WT and BAC rats (Fig. 1i) and reversed enhanced DA turnover (DOPAC + HVA + 3MT/DA) in the hypothalamus of BAC rats (Fig. 1j). In the hippocampus, BAC rats demonstrated decreased noradrenaline (NA) levels (Fig. S4). Overall, CORT administration resulted in anticipated behavioral and neurochemical changes. Several stress endpoints, neurochemical and behavioral outcomes indicate a baseline imbalance of the stress system in BAC animals, rendering them more sensitive to the effects of CORT.

### Chronic corticosterone enhances asyn pathology in the hypothalamus of asyn BAC rats

**Fig. 2:**
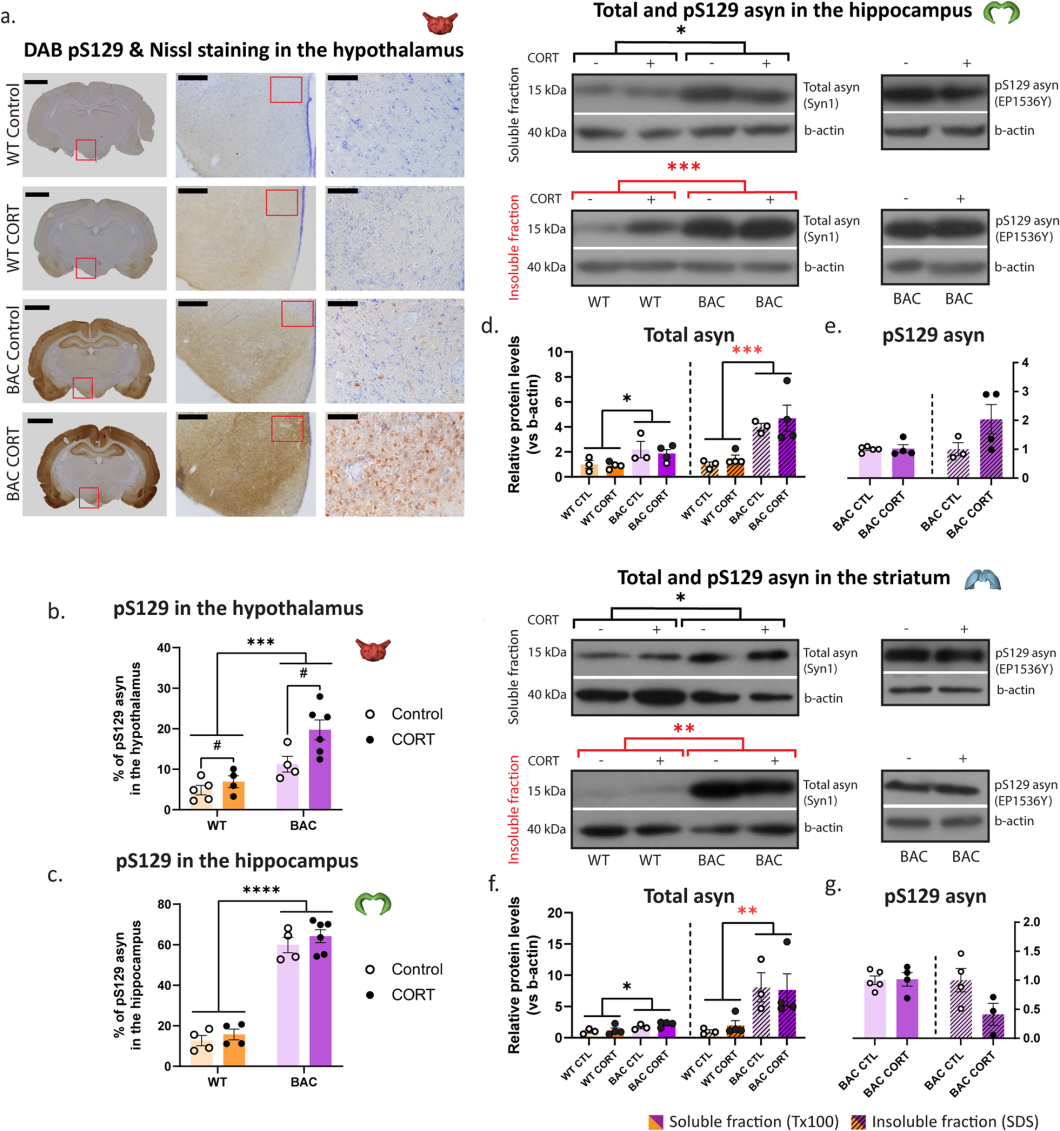
PS129 asyn pathology in stress-related brain regions. Asyn pathology was assessed in BAC rats following chronic CORT with **a** DAB pS129 asyn staining in the hypothalamus and hippocampus, where we calculated **b**, **c** the % of pS129 asyn, respectively; scale bars; 1st column: 3 mm, 2nd column (× 10): 300 μm and 3rd column (× 40): 75 μm). Immunoblotting quantification of total and pS129 asyn in the soluble and insoluble fractions of the hippocampus and the striatum are presented in Fig. S5. In the hippocampus, we measured the relative protein levels (vs β-actin) of **d** total soluble, total insoluble, **e** pS129 soluble and pS129 insoluble asyn. In the striatum, we measured the relative protein levels of **f** total soluble, total insoluble, **g** pS129 soluble and pS129 insoluble asyn. The relative quantification of protein levels was performed with the use of Fiji/ImageJ. Two-way ANOVAs were applied for **b** and **c** with Bonferroni's multiple comparisons post-hoc tests while unpaired t-tests were applied for the comparisons of the normalized amounts of total and pS129 asyn between the soluble and the insoluble fractions in **d**–**g**. All data are expressed as mean ± SEM. Asterisk (*) is used to mark genotype effects while hashtag (#) marks treatment effects. Significance levels: */# *p* < 0.05; ** *p* < 0.01; *** *p* < 0.001; **** *p* < 0.0001. N = 4–5 for **b** and **c** and N = 3–4 per group for **d**–**g**

The hypothalamus acts as a link between the central nervous and the endocrine systems. Immunohistochemical staining in the hypothalamus (Fig. 2a) revealed extensive pS129 asyn pathology in BAC animals (Fig. 2b) which colocalizes with dopaminergic neurons (Fig. S1 for indicative images of pS129 asyn colocalization with dopaminergic neurons in the hypothalamus).

In the hippocampus, BAC transgenic animals displayed approximately five-fold greater amounts of pS129 asyn than WT animals, regardless of CORT treatment, indicating a potential plateau effect (Fig. 2c). Moreover, Western immunoblot analysis showed elevated soluble and insoluble total asyn levels in the hippocampus of BAC animals, regardless of treatment (Fig. 2d, S5a,e). PS129 asyn levels in the BAC animals remained unchanged in the hippocampus after CORT treatment, in both the soluble and insoluble fractions (Fig. 2e, S5b,f). Comparing the shift in asyn species (insoluble/soluble fraction), there is an overall trend for increased total (Fig. S5i; *p* = 0.0939) and pS129 (Fig. S5j; *p* = 0.0505) asyn levels in BAC animals following CORT.

Analysis in the striatum revealed increased soluble and insoluble total asyn levels in the BAC animals, regardless of treatment (Fig. 2f, Fig. S5c,g). Comparing the shift in asyn species (insoluble/soluble fraction), BAC rats demonstrated increased total asyn levels with an interaction effect, indicating an increase in total asyn levels between WT CTL and BAC CTL groups (Fig. S5k). PS129 asyn levels in the striatum were unchanged in BAC animals upon CORT treatment, in both the soluble and insoluble fractions, (Fig. 2g, S5h). No further differences were observed with respect to the shift in pS129 asyn species (insoluble/soluble fraction) (Fig. S5l). Taken together, our results show that chronic CORT administration significantly enhances pS129 asyn levels in the hypothalamus, along with an apparent shift to more insoluble pS129 asyn in the hippocampus of BAC rats.

### Chronic CORT enhances asyn phosphorylation and worsens nigrostriatal neurodegeneration in asyn BAC rats

**Fig. 3:**
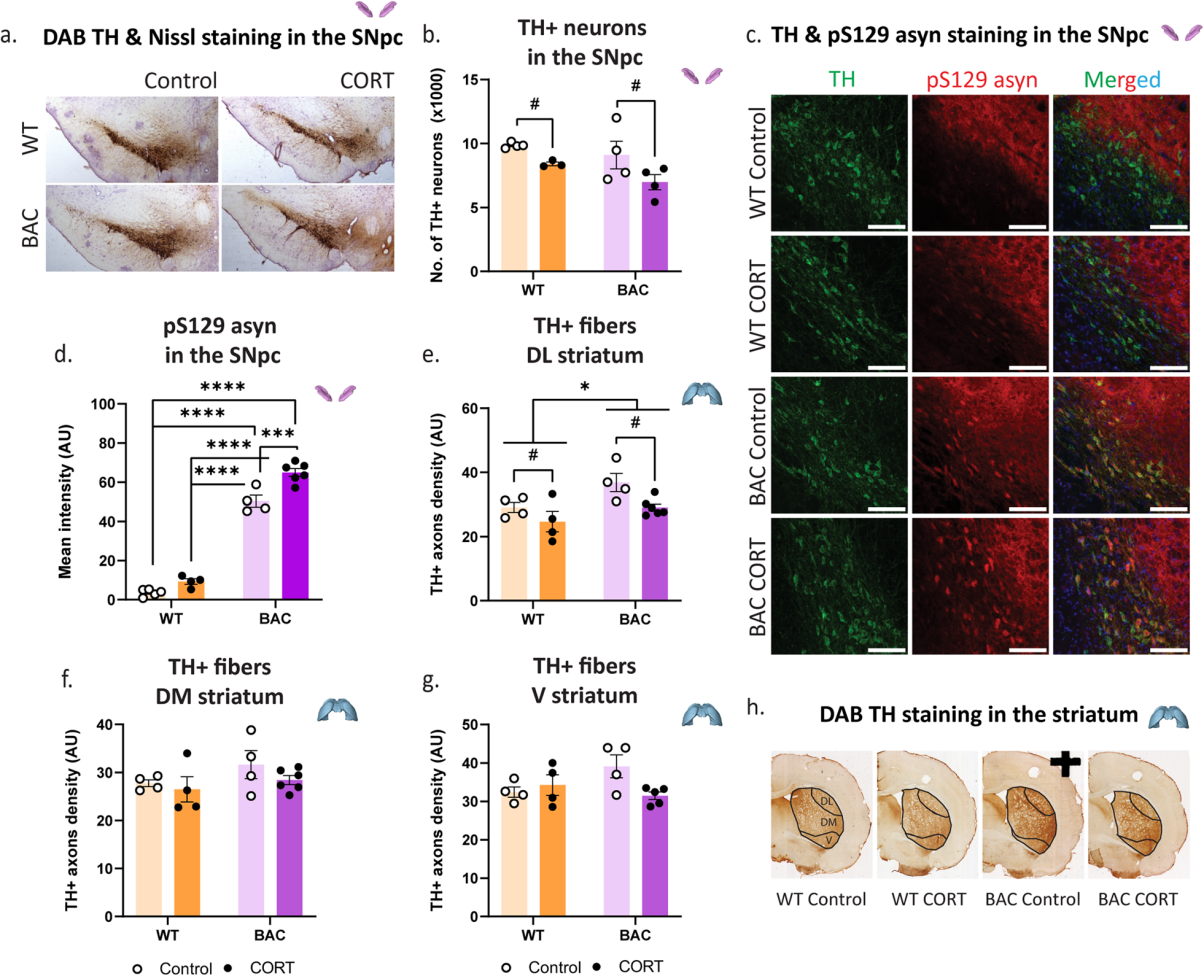
Nigrostriatal dopaminergic system integrity following chronic CORT administration. Nigrostriatal DA system integrity following chronic CORT administration was assessed with **a** DAB TH and Nissl staining in the SNpc to **b** calculate the number of TH + neurons (× 1000). Following **c** immunofluorescent staining against TH and pS129 asyn (scale bar: 100 μm) with DAPI in the column with the merged images, we measured **d** the mean intensity (AU) of pS129 asyn in TH + neurons. **e** TH + axons density in the DL striatum, **f** DM striatum and **g** V striatum were calculated following **h** DAB TH staining. Two-way ANOVAs were applied for **b** and **d**–**g** with Bonferroni's multiple comparisons post-hoc tests. All data are expressed as mean ± SEM. Asterisk (*) is used to mark genotype main effects while hashtag (#) marks treatment main effects. Significance levels: */# *p* < 0.05;*** *p* < 0.001; **** *p* < 0.0001. N = 3–4 per group for **b**, N = 4–6 for **b** and **d**–**g**

Brains were collected and processed to evaluate the integrity of the dopaminergic system by quantifying the TH + neurons in the SNpc (Fig. 3a,b) and amount of pS129 asyn in these cells (Fig. 3c,d), as well as the density of their afferents in the striatum (Fig. 3e–h). Chronic CORT administration led to a significant loss of TH + neurons in the SNpc in both WT and BAC animals, with a loss ranging from 15% for the former to 30% for the latter (Fig. 3b), indicating an additive effect on nigral DA cell loss in BAC rats. Immunohistochemical staining in the SNpc revealed a significant increase of pS129 asyn localized in the TH + cells of the BAC animals, while chronic CORT further increased pS129 asyn levels (significant genotype x treatment interaction), indicating a synergistic effect (Fig. 3d). Separate analysis of the three striatal subdivisions—DL, DM and V striatum—revealed a significantly decreased density of TH + afferents only in the DL striatum following CORT (Fig. 3e). While there were no differences observed in the DM striatum (Fig. 3f), the genotype x treatment interaction effect was significant in the V striatum (Fig. 3g).

In the SNpc (Fig. S6a), immunohistological evaluation of neuroinflammation, revealed a significant increase in the mean intensity of astrocytic (GFAP) cells following chronic CORT administration (Fig. S6b), while no changes were observed in the mean intensity of microglial (Iba1) cell profiles (Fig. S6c). In the striatum, a specific expression pattern for GFAP emerged; immunoblot analysis of the soluble protein fraction (Fig. S6d) demonstrated a significant genotype x treatment interaction effect on GFAP protein expression levels (Fig. S6e). This GFAP expression pattern was further supported by immunohistochemical glial staining in the striatum (Fig. S6f), which also revealed a significant genotype x treatment interaction effect on GFAP mean intensity (Fig. S6g). Iba1 mean intensity in the striatum also displayed a genotype x treatment interaction effect (Fig. S6h) and had a similar expression pattern to GFAP (Fig. S6g). Overall, the glial expression pattern was characterized by significantly increased striatal GFAP mean intensity in the striatum of BAC rats, combined with a genotype x treatment interaction effect, suggesting that BAC rats have already reached a plateau immune activation, achieved by WT animals after CORT administration. The same pattern was seen in the Iba1 mean intensity levels in the striatum.

### Chronic CORT triggers phenoconversion in asyn BAC rats, worsening their Parkinsonian phenotype

**Fig. 4:**
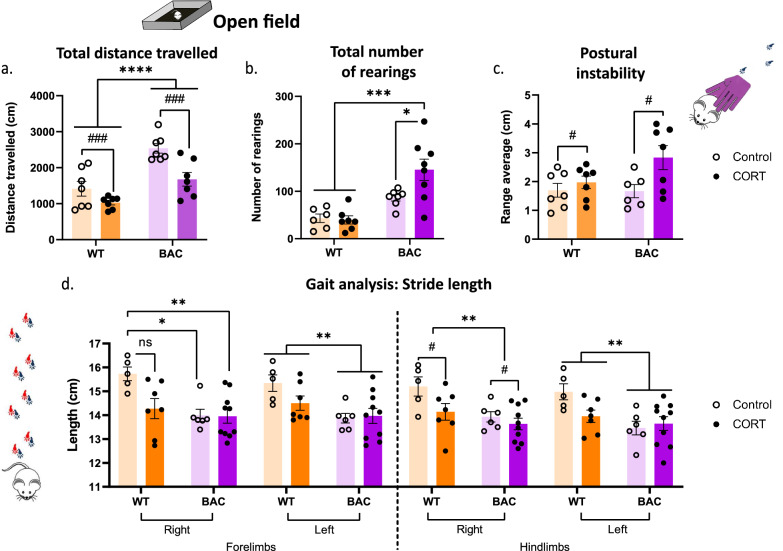
Motor phenotype decline after chronic CORT treatment. Behavioral testing in BAC rats following CORT administration measured **a** the total distance travelled (cm) and **b** the total number of rearings in the OF test. Fine motor function behavioral tests measured **c** the average range (cm) of forelimb steps in the postural instability test and **d** the stride length (cm) both for the forelimbs and the hindlimbs. Two-way ANOVAs were applied for **a**–**d** with Bonferroni's multiple comparisons post-hoc tests. All data are expressed as mean ± SEM. Asterisk (*) is used to mark genotype main effects while hashtag (#) marks treatment main effects. Significance levels: */# *p* < 0.05; ** *p* < 0.01; ***/### *p* < 0.001; **** *p* < 0.0001. N = 5–10 for **a**–**d**

Assessment of gross motor function supported the observed degeneration of dopaminergic neurons in the SNpc following CORT (Fig. 3b). BAC rats exhibited a characteristic baseline locomotor hyperactivity in the open field, expressed as increased distance traveled (cm) (Fig. 4a), while CORT administration decreased motor activity in WT rats and reversed hyperactivity in BAC rats (Fig. 4a).

In addition, a significant increase in the total number of rearings was observed in BAC rats receiving CORT, indicative of augmented exploratory activity (Fig. 4b) and consistent with the hypervigilant state observed in the PPI test (Fig. 1h). Examination of fine motor function revealed that CORT triggered worse performance in the postural instability test for both genotypes (Fig. 4c). Moreover, recorded stride lengths of both forelimbs and hindlimbs revealed CORT—and genotype—dependent changes in gait; decreased right and left forelimb stride length (BAC animals have a shorter stride length, regardless of treatment) and shorter right and left hindlimb stride length (Fig. 4d). The general pattern in these fine motor tasks was for CORT to worsen performance in the WT, but in certain cases, it led to further exacerbation of BAC rats’ gross and fine motor performance.

### Hippocampal stress signaling is perturbed in asyn BAC rats and PD patients

**Fig. 5:**
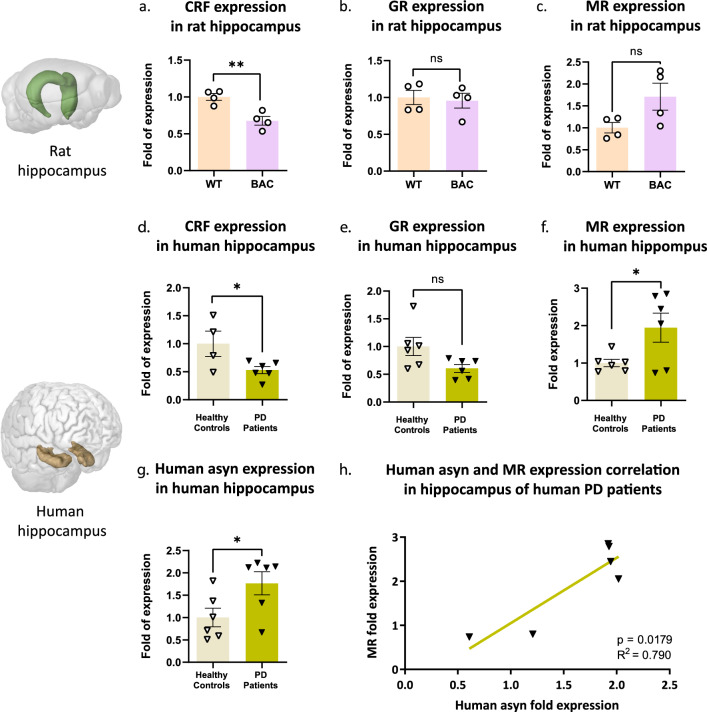
Perturbed hippocampal stress signaling in the brains of human asyn BAC rats and PD patients. In rat hippocampi, we compared the expression levels (fold change) of **a** CRF, **b** GR and **c** MR mRNA between WT and BAC rats. The same measurements were repeated in the human post-mortem hippocampi samples with the calculation of the expression levels (fold change) of **d** CRF, **e** GR, **f** and MR between healthy controls and PD patients. In addition, we examined **g** human asyn gene expression levels between healthy controls and PD patients and **h** the correlation of human asyn with MR gene expression levels of PD patients. Images credit: Allen Institute for Brain Science. Unpaired t-test was applied for **a**–**f** while simple linear regression analysis was applied for **g**. All data are expressed as mean ± SEM. Significance levels: *p < 0.05; ** *p* < 0.01. N = 4 for **a**–**c** and N = 4–6 per group for **d**–**h**

Hippocampal stress signaling is dependent on CRF and hippocampal mineralocorticoid (MR) and glucocorticoid (GR) receptors regulate HPA axis via glucocorticoid positive and negative feedback, respectively. We found that hippocampal CRF expression is diminished in BAC rats (Fig. 5a), as in the hypothalamus (Fig. 1b). Furthermore, hippocampal GR expression remains stable in BAC rats (Fig. 5b), while there is a nonsignificant increase in MR receptor expression (Fig. 5c; *p* = 0.0755). To ascertain if hippocampal stress signaling is affected in PD patients, RNA was extracted from human post-mortem hippocampal samples derived from healthy controls and PD patients. We demonstrated decreased CRF mRNA levels (Fig. 5d), nonsignificantly decreased GR gene expression levels (Fig. 5e; *p* = 0.0512), and increased MR gene in PD patients (Fig. 5f). Importantly, asyn expression levels in the hippocampus of PD patients were significantly elevated (Fig. 5g) and strongly correlated with MR expression levels (Fig. 5h). Collectively, our results demonstrate that in the presence of excess asyn, in BAC rats and PD patients, glucocorticoid signaling is altered, indicating baseline stress sensitivity.

## Discussion

There is a relative lack of depth in preclinical research assessing HPA axis pathology in PD [12, 23], despite the high comorbidity between PD and depression, an established stress-system disorder [24]. In this study, we provide the first evidence for asyn-linked HPA axis hypoactivation and aberrant brain stress signaling in PD, indicative of glucocorticoid/stress hypersensitivity [25, 26]. In parallel, glucocorticoid hypersensitivity in asyn BAC rats leads to enhanced expression of pS129 asyn in the hypothalamus and in dopaminergic neurons of the substantia nigra upon chronic CORT administration, suggesting direct effects of circulating glucocorticoids on asyn pathology. These results explain at least in part, the ability of chronic stress to trigger phenoconversion from a prodromal to a more overt motor phenotype, associated with astrocytic activation in the striatum and aggravated nigrostriatal neurodegeneration. Finally, asyn-associated decreased CRF gene expression observed in the hippocampi of both asyn BAC rats and PD patients also supports stress hypersensitivity. Taken together, our results link asyn with brain stress system hypoactivation/dysregulation that confers enhanced sensitivity to stress system activation; thus, elevated circulating glucocorticoids can impact already offset brain stress homeostatic mechanisms, potentially triggering, or at least contributing to, PD-related neurodegeneration and emergence of clinical symptoms.

### Overexpression of human wildtype asyn leads to baseline HPA *axis* dysregulation

The most direct measurement of HPA axis function in humans is blood cortisol levels. The directionality of these changes in PD is inconclusive with reports of decreased [27, 28], increased [29–31] or unaltered [32, 33] cortisol levels. These differences can be explained by the lack of large-scale, multi-sampled, circadian studies up until today and variable inclusion criteria, including age, drug treatment and disease stage [12]. Interestingly, the COVID-19 pandemic, considered as an environmental stressor, did not accelerate PD progression, but exacerbated depressive symptoms in stressor-reactive (vs. stressor-resilient) PD patients [34].

The hypothalamus is a highly-conserved brain region involved in the regulation of core homeostatic processes, neuroendocrine integration and autonomic output required for an appropriate stress response. Asyn is present in the hypothalamus of PD patients [35, 36] and Lewy bodies have been detected in all hypothalamic nuclei [37]. In line with our findings (Fig. 2a,b), enhanced hypothalamic pS129asyn was observed in Thy1-asyn mice [38]. pS129asyn affects asyn structure and aggregation propensity [39] and is used as a surrogate marker of asyn aggregation and toxicity [40], despite the ongoing debate as to whether this post-translational modification is associated with neurotoxicity or neuroprotection [41]. Furthermore, decreased CRF-like immunoreactivity has been observed in post mortem cortical tissue from PD brains [42] and MPTP treatment in mice leads to selective reduction of CRF-positive neurons in the PVN, suggesting that hypothalamic CRF neuron integrity is regulated by dopaminergic pathways [12]. Indeed, we observed robust expression of pS129 asyn in dopaminergic neurons of the PVN (Fig. S1). Enhanced hypothalamic DA turnover in BAC rats (Fig. 1j)—previously reported in the striatum [14]—is indicative of compensatory mechanisms also reported in patients [43]. Interestingly, CORT completely reversed this increase, suggesting that it constitutes a tipping point, leading to phenoconversion.

A critical regulator of HPA axis function is the hippocampus [44, 45], a brain region with abundant asyn expression [14, 46]. Depressed PD patients’ hippocampal volumes are inversely correlated with their clinical depression scores [47]. Moreover, HPA axis function is affected in PD and together, these changes may help explain in part the high comorbidity with depression and anxiety (22 and 25–40%, respectively) [48]. Finally, besides CRF’s well-known role in mediating HPA axis function, hippocampal stress signaling and neurogenesis also require intact CRF signaling [49, 50].

Our present findings of (1) enhanced pS129 asyn (particularly in DA-expressing neurons) in the hypothalamus (Fig. 2a,b), (2) an apparent shift to more insoluble pS129 in the hippocampus (Fig. 2a,c, S5j), (3) decreased CRF expression in both regions (Fig. 1b, 5a), (4) decreased baseline plasma CORT (Fig. 1c) and (5) adrenal gland atrophy (Fig. 1d), indicate multi-level HPA axis dysfunction, including partial adrenal insufficiency associated with asyn-dependent PD pathology. We propose that asyn-dependent baseline HPA axis dysregulation leads to enhanced stress system sensitivity/responsivity, consistent with the observed anxiety-like phenotype (Fig. 1e, h, 4b) in asyn BAC rats. Interestingly, a transcriptomic analysis in healthy control subjects revealed a four-fold increase in asyn expression in glucocorticoid-sensitive subjects vs. glucocorticoid-resistant subjects [51]. Presumably, baseline HPA axis dysregulation and stress hyper responsivity sets the stage for enhanced stress sensitivity, i.e. via altered stress signalling (discussed below), and subsequent neurodegeneration/phenoconversion upon exposure to chronic stress.

### Chronic corticosterone leads to phenoconversion from prodromal to overt motor PD

Chronic stress is a detrimental environmental factor that leads to sustained hyper- or hypo- activation of the HPA axis, glucocorticoid hyper- or hypo- secretion, decreased DA levels and increased DA turnover in the hippocampus [52, 53]. In rodent models, chronic stress is associated with increased anxiety and depressive-like behaviors [54–57] and in humans, chronic stress is a major risk factor for accelerating the onset of and exacerbating symptomatology of depression and anxiety, although there is no clear link between the existence of these comorbidities with PD and accelerated or worse clinical outcome (reviewed in [12, 23]). Chronic emotional stress per se may cause dopaminergic neurodegeneration in susceptible individuals [9] and rodents [11], and is known to accelerate dopaminergic neurodegeneration, subsequently exacerbating motor symptoms in toxin models of PD [10, 58]. Furthermore, chronic CORT administration reduced proteostatic stress adaptation to intrastriatal asyn preformed fibril injections, lowering the threshold for asyn pathology and nigral neurodegeneration [13] and chronic mild stress was associated with microglial activation and cytokine release, midbrain asyn inclusion pathology, exacerbated nigrostriatal neurodegeneration, and motor worsening in A53T male mice [11]. Importantly, the present findings confirm the neurodegenerative potential of chronic stress to accelerate asyn pathology and nigrostriatal neurodegeneration and extend them by demonstrating its potential to act as a trigger of phenoconversion.

Patients with preclinical PD may demonstrate symptoms characteristic of severe DA deficiency following stress [59] indicating temporary stress-induced acceleration of the disease [60, 61]. We previously reported mild nigrostriatal neurodegeneration along with increased striatal DA, motor hyperactivity and other non-motor symptoms in asyn BAC rats; a phenotype resembling prodromal PD [14]. Chronic CORT decreased dopaminergic cell numbers and fibers in the substantia nigra and the anatomically-connected DL striatum, respectively (Fig. 3b,e). These results corroborate with neuroanatomical findings that dopaminergic neurons located in the SNpc with axonal projections to the DL striatum are preferentially degenerated in PD [62]. Additionally, the increased density of TH + fibers observed in the BAC rats (Fig. 3e), is in line with striatal hyperdopaminergia we previously reported [14]. CORT appears to act as a facilitator or aggravator of PD pathogenesis as demonstrated by its “phenoconverting” ability to reverse baseline motor hyperactivity (Fig. 4a) [14] and increased striatal dopaminergic fiber density in asyn BAC rats (i.e. compensatory enhanced dopaminergic signalling in the striatum; Fig. 3e). In parallel, subtle fine motor deficits manifested upon chronic stress exposure, including worsening of postural instability (Fig. 4c) and decreased right hindlimb stride length (Fig. 4d). Thy1-aSYN mice seem to be the only transgenic PD mice that naturally phenoconvert with age—their natural history evolves from a state of increased extracellular striatal DA at 6 months of age to decreased locomotion at 14 months of age, when striatal DA is significantly depleted [63, 64]. Here, we report an accelerated model of phenoconversion to a neurodegenerative state in 9 mo asyn BAC rats using chronic CORT, demonstrating the importance of gene-environment interactions in the study of PD pathogenesis.

We also observed a reversal of enhanced DA turnover in the hypothalamus of asyn BAC rats following chronic CORT treatment (Fig. 1j), further supporting phenoconversion and apparent baseline dysregulation of HPA axis at the neurotransmission level as well. 18 Fluoro-dopa Positron Emission Tomography has demonstrated reduced monoamine storage capacity in PD [65], thus, enhanced DA turnover may be a consequence of reduced storage capacity in asyn BAC rats and chronic CORT may alter the metabolic activity of dopaminergic neurons, decreasing their turnover.

Neuroinflammation has a cause-effect relationship with PD. Nigrostriatal astrocytic activation is in accordance with dopaminergic neuron death [66–68]. Reactive microglia are detected in the hippocampus of PD brains [69, 70] and loss of GR signalling in the substantia nigra of MPTP-treated mice and PD patients is caused by nuclear re-localization of GR in activated microglia, demonstrating their pivotal role in regulating dopaminergic neurodegeneration [71]. Microglial activation was not as prominent although it followed the same pattern as astrocytic activation (Fig. S6). The robust decrease in nigral dopaminergic cell number (Fig. 3b), decreased locomotor activity (Fig. 4a) and fine motor deficits (Fig. 4c,d) – a profile matched by WT only after CORT treatment – indicates that, temporally, inflammation precedes dopaminergic cell loss.

### Hippocampal markers of stress signalling are perturbed in asyn BAC rats and these changes are conserved in human PD brains

Asyn inclusions are abundant in the hippocampus and associate with cognitive dysfunction, especially in later stages of the disease [72–75] and asyn expression is greatest in the hippocampus in rodent overexpression models [14, 76]. Additionally, BAC rats were previously reported to have severely impaired neurogenesis and anxiety-like behavior [77] while CRF was recently identified as a local factor mediating neuronal stem cell proliferation and survival in adult hippocampal neurogenesis [50]. With regards to CRF’s critical role in cognitive processes [78], decreased hippocampal CRF expression observed in both BAC rats (Fig. 5a) and PD patients (Fig. 5d) may provide a direct link between hippocampal stress system dysfunction and comorbid anxiety in PD.

Hippocampal stress signalling is also modulated by GRs and MRs that exercise respectively negative and positive feedback on HPA axis function by detecting levels of circulating glucocorticoids, thereby extinguishing the stress response [79]. MRs have a tenfold greater affinity for CORT and are fully occupied by glucocorticoids at baseline [80, 81] while GRs are activated when circulating CORT levels are elevated during stress [82–84]. GR and MR expression is most abundant in the hypothalamus and hippocampus, in both humans and rodents [85, 86].

Previous studies in animal models or PD patients, demonstrate that chronically high levels of glucocorticoids compromise immune function and result in GR downregulation [30, 87]. GR dysfunction occurs over time, leading to DA neuronal loss and clinical manifestation of PD [88]. Generally, GR is associated with an anti-inflammatory role, and MR with a pro-inflammatory role [87]. Thus, observed GR downregulation and MR upregulation (Fig. 5b,c,e,f) results in downregulation of the stress response and is conducive to a pro-inflammatory and neurodegenerative status—potentially rendering the asyn PD brain hypersensitive to elevated glucocorticoid levels and subsequent deficit in coping with chronic stress exposure. The constellation of GR/MR imbalance, HPA axis dysregulation, and anxiety-like phenotype of BAC rats (Fig. 1e,h), provide insight into a potential mechanism of comorbid depression and anxiety in PD.

Finally, conserved GR/MR imbalance and MR upregulation correlated with asyn levels (Fig. 5h) in post mortem PD brains provides a first glimpse of perturbed molecular stress signalling, associated with asyn in PD patients. Thus, asyn pathology increases the brain’s allostatic and as the disease progresses, enhancing its susceptibility to chronic stress and accelerating its detrimental consequences.

Limitations of the current study include the stress paradigm examined, chronic CORT vs. chronic unpredictable stress, which is considered more representative of real-world chronic stress conditions in humans. Importantly, potential sex differences in stress-gene interactions involved in phenoconversion need to be pursued in follow-up studies, considering that females: (1) appear to be more protected to environmental stressors like rotenone [89], (2) show a differential hippocampal response to corticosterone [90], and (3) higher overall susceptibility to stress-related disorders [52, 91]. Finally, in the present study, stress signaling perturbations are observed in PD patients with advanced stage PD and our findings are limited to the hippocampus, whilst it would be interesting to probe the nigrostriatal pathway in future studies.

## Conclusions

This study demonstrates HPA axis dysregulation prior to overt neurodegeneration in human-asyn BAC overexpression transgenic rats. The animal data are supported by analogous stress signalling changes in post-mortem hippocampi of PD patients, suggesting that PD patients have a greater sensitivity to stress, which positively correlates with asyn expression. Chronic corticosterone administration, by enhancing asyn pathological changes, appears to override compensatory mechanisms leading to more overt neurodegeneration and subsequent emergence of Parkinsonian phenotypes. Thus, under conditions of enhanced asyn burden/pathology, chronic stress as an environmental factor can potentially “trigger” PD phenoconversion; chronic stress protocols could therefore be used as critical research tools to study the underlying mechanisms of phenoconversion in PD.

### Supplementary Information

**Additional file 1: Table S1.** Demographic details for healthy control (N=6) and PD (N=6) cohorts.

**Additional file 2: Table S2.** Lists of primary and secondary antibodies.

**Additional file 3: Figure S1.** Immunofluorescence staining against TH and pS129 asyn in the hypothalamus.** a** In the pictures that were captured with the 40x 1.20 objective, the scale bar is 50 μm and** b** in the pictures that were captured with the 63x 1.30 objective, the scale bar is 10 μm.

**Additional file 4: Table S3.** List of RT qPCR primers.

**Additional file 5: Table S4.** Exact p-values from the analyses of the experiments presented in Figures and Additional files.

**Additional file 6: Figure S2.** Total distance travelled (cm) in the EPM by WT and BAC animals after two weeks of CORT administration. Two-way ANOVA was applied with Bonferroni’s multiple comparisons post-hoc tests. Asterisk (*) is used to mark genotype effects while hashtag (#) marks treatment main effects. All data are expressed as Mean ± SEM. Significance levels: # p < 0.05; *** p < 0.001. N = 7–8.

**Additional file 7: Figure S3.** Simple linear regression correlation analysis of the time (s) spent in the center of the OF arena to the total distance travelled (cm) in the OF arena for the animals of all the experimental groups, regardless of genotype and treatment group. Linear regression analysis was applied. N = 26.

**Additional file 8: Figure S4.** NA levels (mg/g wet tissue) in the hippocampus of WT and BAC animals after two weeks of CORT administration. Two-way ANOVA was applied with Bonferroni’s multiple comparisons post-hoc tests. All data are expressed as Mean ± SEM. Asterisk (*) is used to mark genotype effects. Significance levels: **p < 0.01. N = 3–4.

**Additional file 9: Figure S5.** By immunoblot biochemical analysis we measured relative protein levels (vs b-actin) of** a** total asyn levels in the soluble fraction of hippocampus,** b** pS129 asyn levels in the soluble fraction of hippocampus,** c** total asyn levels in the soluble fraction of striatum,** d** pS129 asyn levels in the soluble fraction of striatum,** e** total asyn levels in the insoluble fraction of hippocampus,** f** pS129 asyn levels in the insoluble fraction of hippocampus** g** total asyn levels in the insoluble fraction of striatum and** h** pS129 asyn levels in the insoluble fraction of striatum. We calculated insoluble to soluble fraction ratios of** i** total asyn levels in hippocampus,** j** pS129 asyn levels in hippocampus,** k** total asyn levels in striatum and** l** pS129 asyn levels in striatum. The relative quantification of protein levels was performed with the use of Fiji/ImageJ. Two-way ANOVAs were applied for (**a**), (**c**), (**e**), (**g**), (**i**) and (**k**) with Bonferroni’s multiple comparisons post-hoc tests while unpaired t-tests were applied for the comparisons (**b**), (**d**), (**f**), (**h**), (**j**) and (**l**). Asterisk (*) is used to mark genotype effects. All data are expressed as Mean ± SEM. Significance levels: * p < 0.05; ** p < 0.01; *** p < 0.001. N = 3-5.

**Additional file 10: Figure S6. a** Following immunohistochemical staining in the SNpc we calculated the mean intensity (AU) of** b** astrocytes (GFAP) and** c** microglia (Ibal) expression.** d** By immunoblot biochemical analysis we measured** e** relative protein levels (vs GAPDH) of GFAP in the soluble fraction of the striatum.** f** By applying immunohistochemical staining in the striatum we calculated the mean intensity (AU) of** g** astrocytes (GFAP) and** h** microglia (Ibal) expression. The relative quantification of protein levels was performed with the use of Fiji/ImageJ. Regular 2-way ANOVAs were applied for (**b**), (**c**), (**e**), (**g**) and (**h**) with Bonferoni's multiple comparisons post-hoc tests. Aserisk (*) is used to mark genotype main effects while hashtag (#) marks treatment main effects. All data are expressed as Mean ± SEM. Significant levels: */# p < 0.05, ** p < 0.01. N = 4–6.

## Data Availability

All the raw data that support the findings of this study are available from the corresponding author, upon request.

## Abbreviations

3MT: 3-Methoxytyramine
Asyn: Alpha-synuclein
AU: Arbitrary units
BAC: Bacterial artificial chromosome transgene (overexpressing the human full-length wildtype alpha-synuclein protein)
CORT: Corticosterone
CRF: Corticotropin releasing factor
DA: Dopamine
DAB: Diaminobenzidine
DAPI: 4′,6-Diamidino-2-phenylindole
DL: Dorsolateral
DM: Dorsomedial
DOPAC: 3,4-Dihydroxyphenylacetic acid
EPM: Elevated plus maze
GFAP: Glial fibrillary acidic protein
GR: Glucocorticoid receptor
HPA: Hypothalamic–pituitary–adrenal
HVA: Homovanillic acid
Iba1: Ionized calcium binding adaptor molecule 1
MR: Mineralocorticoid receptor
NA: Noradrenaline
PD: Parkinson’s disease
PPI: Prepulse inhibition
pS129: Serine 129 phosphorylation
SDS: Sodium dodecyl sulfate
SNpc: Substantia nigra pars compacta
TH: Tyrosine hydroxylase
Tx100: Triton-x-100
V: Ventral
