# Supplementary material for: Alpha-synuclein-induced stress sensitivity renders the Parkinson’s disease brain susceptible to neurodegeneration
Source: Acta Neuropathol Commun. 2024 Jun 17;12:100. doi: 10.1186/s40478-024-01797-w (PMC11181569; doi:10.1186/s40478-024-01797-w)
Supplement: Supplementary file 1 — Additional file 1: Table S1. Demographic details for healthy control (N=6) and PD (N=6) cohorts. [file 40478_2024_1797_MOESM1_ESM.pdf]

**Additional file 1: Table S1.** Demographic details for healthy control (N=6) and PD (N=6) cohorts

| Sample | Age (years) | Onset (years) | Duration (years) | PMD (hours) |
|---|---|---|---|---|
| PD n=6 | 81+/- 3.65 | 65.9 +/-7.99 | 15.1 +/- 5.63 | 16.3 +/- 6.7 |
| Healthy controls n=6 | 82.1 +/- 6.03 | n.a. | n.a. | 18 +/- 6.97 |

n.a., not applicable
