# Supplementary material for: Alpha-synuclein-induced stress sensitivity renders the Parkinson’s disease brain susceptible to neurodegeneration
Source: Acta Neuropathol Commun. 2024 Jun 17;12:100. doi: 10.1186/s40478-024-01797-w (PMC11181569; doi:10.1186/s40478-024-01797-w)
Supplement: Supplementary file 2 — Additional file 2: Table S2. Lists of primary and secondary antibodies. [file 40478_2024_1797_MOESM2_ESM.pdf]

Additional file 2: Table S2. Lists of primary and secondary antibodies

Primary Antibody against	Clone	Catalog number	Working dilutions (WB)	Working dilutions (IHC)	Host species	Company
Actin	C4	MAB1501	1/5000	-	mouse	EMD Millipore
GAPDH	6C5	MAB374	1/5000	-	mouse	EMD Millipore
GFAP (GA5)	-	3670	1/1000	1/1000	mouse	Cell Signaling Technology
Iba1/AIF-1	-	17198	-	1/400	rabbit	Cell Signaling Technology
Phospho S129	EP1536Y	ab51253	1/1000	1/2000	rabbit	Abcam
Total SNCA	SYN1	610786	1/1000	1/1000	mouse	BD Transduction Laboratories
Tyrosine Hydroxylase	LNC1	MAB318	-	1/1000	mouse	EMD Millipore
	-	ab76442	-	1/1000	chicken	Abcam

Secondary antibody	Catalog number	Working dilutions (ICC or WB)	Company
CF488A green	-	1/2000	Biotium
CF555 red	-	1/2000	Biotium
Cy5	128-175-160	1/400	Jackson Imm. Affinipure
