# Supplementary figures and images for: Alpha-synuclein-induced stress sensitivity renders the Parkinson’s disease brain susceptible to neurodegeneration
Source: Acta Neuropathol Commun. 2024 Jun 17;12:100. doi: 10.1186/s40478-024-01797-w (PMC11181569; doi:10.1186/s40478-024-01797-w)

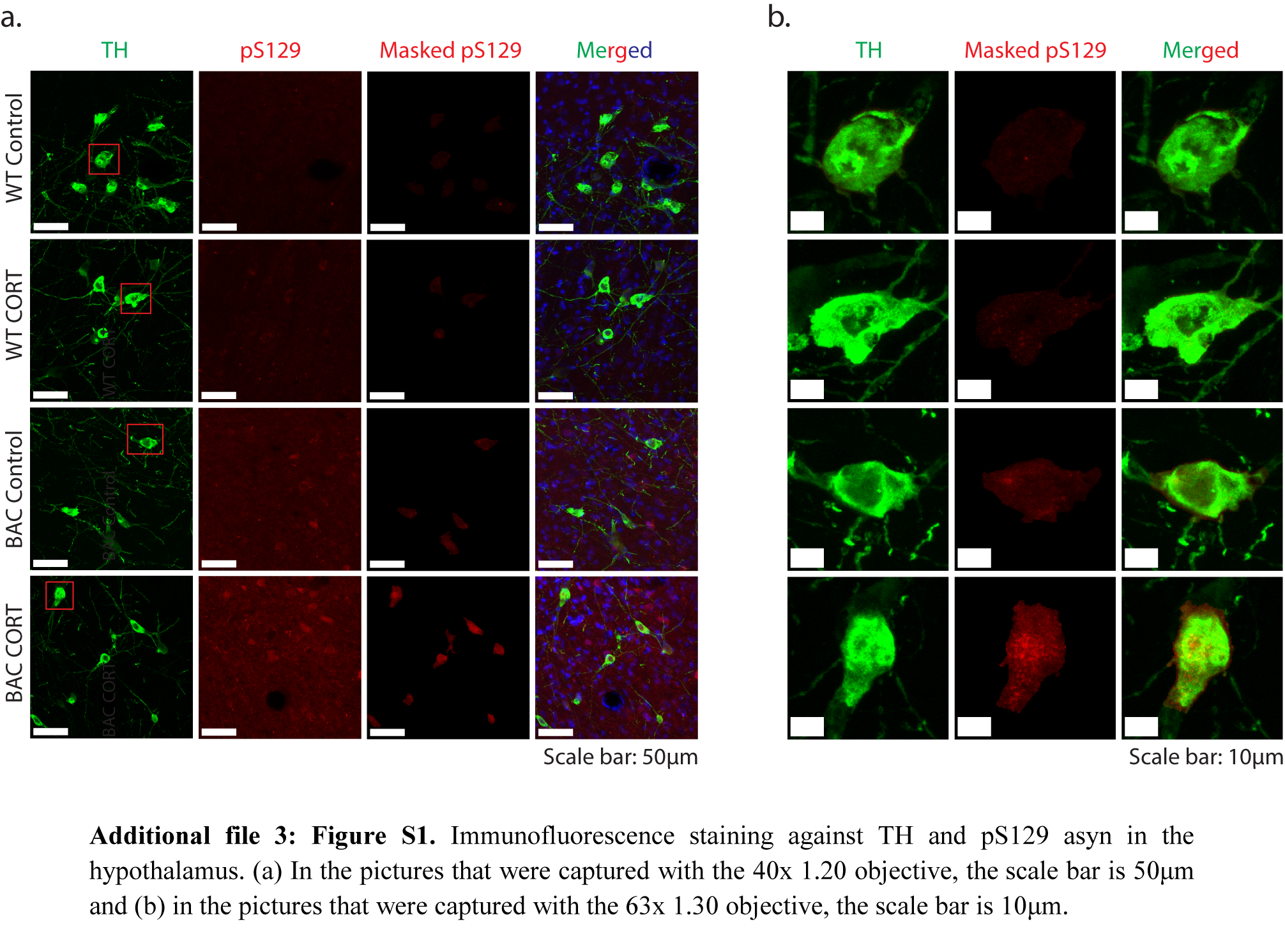

Supplement: Supplementary file 3 — Additional file 3: Figure S1. Immunofluorescence staining against TH and pS129 asyn in the hypothalamus. a In the pictures that were captured with the 40x 1.20 objective, the scale bar is 50 μm and b in the pictures that were captured with the 63x 1.30 objective, the scale bar is 10 μm. [file 40478_2024_1797_MOESM3_ESM.tif]

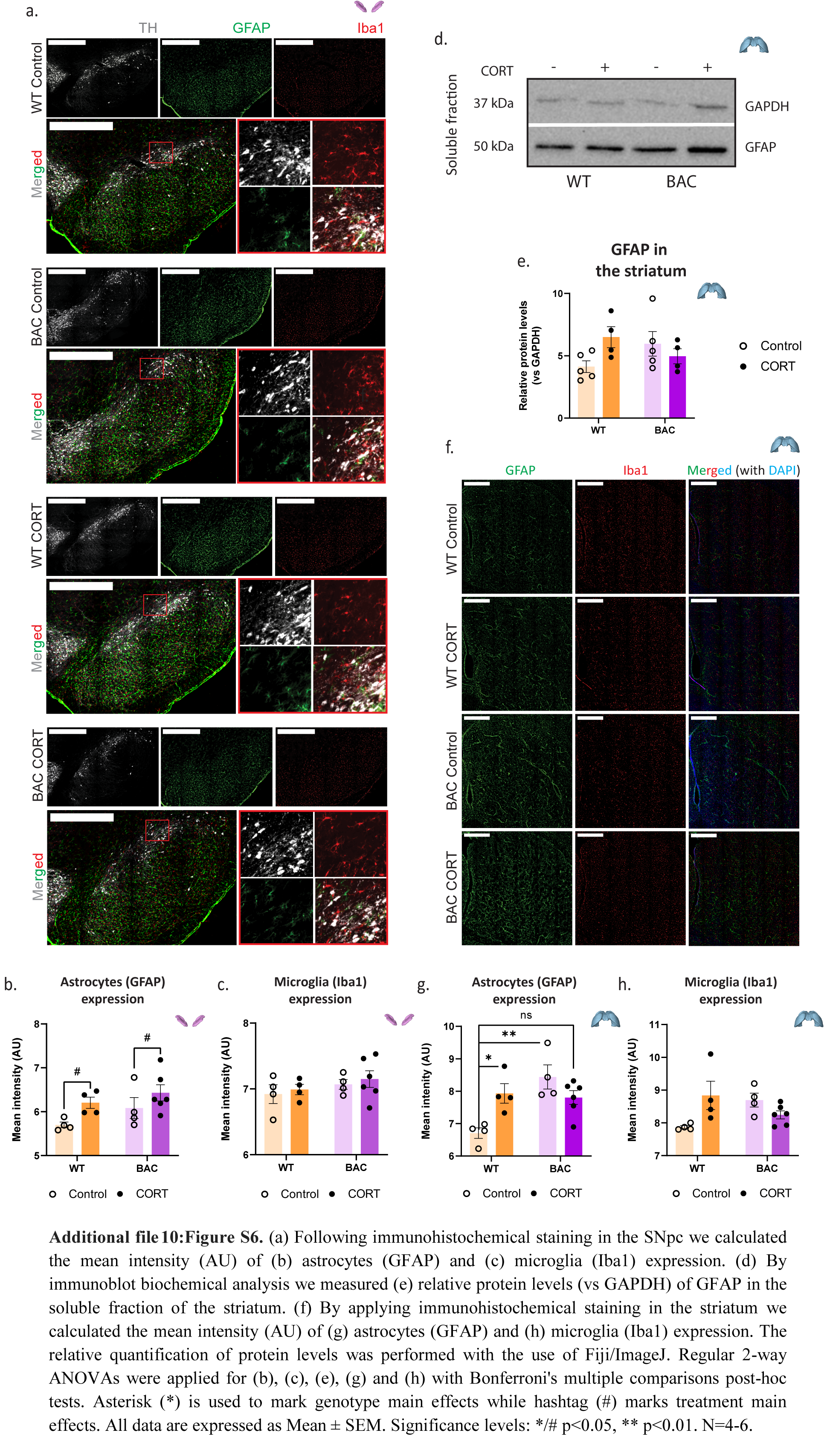

Supplement: Supplementary file 10 — Additional file 10: Figure S6. a Following immunohistochemical staining in the SNpc we calculated the mean intensity (AU) of b astrocytes (GFAP) and c microglia (Ibal) expression. d By immunoblot biochemical analysis we measured e relative protein levels (vs GAPDH) of GFAP in the soluble fraction of the striatum. f By applying immunohistochemical staining in the striatum we calculated the mean intensity (AU) of g astrocytes (GFAP) and h microglia (Ibal) expression. The relative quantification of protein levels was performed with the use of Fiji/ImageJ. Regular 2-way ANOVAs were applied for (b), (c), (e), (g) and (h) with Bonferoni's multiple comparisons post-hoc tests. Aserisk (*) is used to mark genotype main effects while hashtag (#) marks treatment main effects. All data are expressed as Mean ± SEM. Significant levels: */# p < 0.05, ** p < 0.01. N = 4–6. [file 40478_2024_1797_MOESM10_ESM.tif]
