# Supplementary material for: Alpha-synuclein-induced stress sensitivity renders the Parkinson’s disease brain susceptible to neurodegeneration
Source: Acta Neuropathol Commun. 2024 Jun 17;12:100. doi: 10.1186/s40478-024-01797-w (PMC11181569; doi:10.1186/s40478-024-01797-w)
Supplement: Supplementary file 4 — Additional file 4: Table S3. List of RT qPCR primers. [file 40478_2024_1797_MOESM4_ESM.pdf]

Additional file 4: Table S3. List of RT qPCR primers

**Gene**	**Forward (5’→ 3’)**	**Reverse (5’→ 3’)**
Human GR (NR3C1)	ATAGCTCTGTTCCAGACTCAACT	TCCTGAAACCTGGTATTGCCT
Human MR (NR3C2)	AAGTCGTGAAGTGGGCAAAG	CCAAGAATACTGGATTAGGGT
Human GAPDH	CCTCTGACTTCAACAGCGACAC	AGCCAAATTCGTTGTCATACCAG
Human CRF	CATCTCCCTGGATCTCACCTTC	AATAATCTCCATGAGTTTCCTGTTG
Human RPL13A	CCTGGAGGAGAAGAGGAAAGAGA	TTGAGGACCTCTGTGTATTTGTCAA
Human asyn	GCCTCTGACTTCAACAGCGACAC	ATTGATGGGAAGGCATCAGA
Rat GR (NR3C1)	AACGGAGGVAGTGTGAAAT	GGGACTCTCGTTTGTGTCTTTTA
Rat MR (NR3C2)	GTGTGCTGGAAGAAATGAC	CAGCTTCTTTGACTTTCG
Rat CRF	CTCTCTGGATCTCACCTTCCAC	CTAAATGCAGAATCGTTTTGGC
Rat GAPDH	ATGACTCTACCCACGGCAAG	CTGGAAGATGGTGATGGGTT
