# Supplementary material for: Alpha-synuclein-induced stress sensitivity renders the Parkinson’s disease brain susceptible to neurodegeneration
Source: Acta Neuropathol Commun. 2024 Jun 17;12:100. doi: 10.1186/s40478-024-01797-w (PMC11181569; doi:10.1186/s40478-024-01797-w)
Supplement: Supplementary file 5 — Additional file 5: Table S4. Exact p-values from the analyses of the experiments presented in Figures and Additional files. [file 40478_2024_1797_MOESM5_ESM.pdf]

Additional file 5: Table S4. Exact p-values from the analyses of the experiments presented in Figures and Additional files

Figure	Title	Statistical test	P value	P value summary	Significantly different	Multiple comparisons
1b	Corticotropin release factor in the hypothalamus	Unpaired t-test	0.0229	*	Yes	Does not apply
1c	Plasma corticosterone	Two-way ANOVA	Genotype effect: 0.0182<br>Treatment effect: 0.0628	*<br>#	Yes<br>No	-
1d	Adrenal glands weight	Two-way ANOVA	Genotype effect: 0.0342	*	Yes	-
1e	Elevated plus maze	Two-way ANOVA	Interaction effect: 0.0135<br>Genotype effect: 0.0005<br>Treatment effect: 0.0282	*<br>***<br>#	Yes<br>Yes<br>Yes	WT CTL vs WT CORT: p=0.0098 (**)<br>WT CTL vs BAC CTL: p=0.0007 (***)<br>WT CTL vs BAC CORT: p=0.0008 (***)
1f	Time spent in the center of the open field arena	Two-way ANOVA	Genotype effect: 0.0001<br>Treatment effect: 0.0013	**<br>###	Yes<br>Yes	-
1g	Average PPI	Two-way ANOVA	Treatment effect: 0.0075	##	Yes	-
1h	Acoustic startle: Stimulus response	Two-way ANOVA	Interaction effect: 0.0047<br>Genotype effect: 0.0053	**<br>##	Yes<br>Yes	WT CTL vs BAC CORT: p=0.0423 (*)<br>WT CORT vs BAC CORT: p=0.0007 (***)<br>BAC CTL vs BAC CORT: p=0.0532 (ns)
1i	Noradrenaline (NA) in the hypothalamus	Two-way ANOVA	Treatment effect: 0.0029	##	Yes	-
1j	Dopamine (DA) turnover in the hypothalamus	Two-way ANOVA	Interaction effect: 0.0019<br>Genotype effect: 0.0033<br>Treatment effect: 0.0117	**<br>**<br>#	Yes<br>Yes<br>Yes	WT CTL vs BAC CTL: p=0.0031 (**)<br>WT CORT vs BAC CTL: p=0.0027 (**)<br>BAC CTL vs BAC CORT: p=0.0018 (**)
2b	pS129 in the hypothalamus	Two-way ANOVA	Genotype effect: 0.0002<br>Treatment effect: 0.0183	***<br>#	Yes<br>Yes	-
2c	pS129 in the hippocampus	Two-way ANOVA	Genotype effect <0.0001	****	Yes	-
2d	Total asyn in the hippocampus (soluble fraction)	Two-way ANOVA	Genotype effect: 0.0165	*	Yes	-
2d	Total asyn in the hippocampus (insoluble fraction)	Two-way ANOVA	Genotype effect: 0.0008	***	Yes	-
2f	Total asyn in the striatum (soluble fraction)	Two-way ANOVA	Genotype effect: 0.0175	*	Yes	-
2f	Total asyn in the striatum (insoluble fraction)	Two-way ANOVA	Genotype effect: 0.0066	**	Yes	-
3b	TH+ neurons in the SNpc	Two-way ANOVA	Treatment effect: 0.0223	*	Yes	-
3d	pS129 asyn in the SNpc	Two-way ANOVA	Interaction effect: 0.0403<br>Genotype effect<0.0001<br>Treatment effect: 0.0001	*<br>****<br>###	Yes<br>Yes<br>Yes	WT CTL vs BAC CTL: p<0.0001 (****)<br>WT CTL vs BAC CORT: p<0.0001 (****)<br>WT CORT vs BAC CTL: p<0.0001 (****)<br>WT CORT vs BAC CORT: p<0.0001 (****)<br>BAC CTL vs BAC CORT: p=0.0005 (***)

(**Table S4.** continues on the next page)

Table S4. (continued) Exact p-values from the analyses of the experiments presented in Figures and Additional files

Figure	Title	Statistical test	P value	P value summary	Significantly different	Multiple comparisons
3e	TH+ fibers DL striatum	Two-way ANOVA	Genotype effect: 0.0138<br>Treatment effect: 0.0137	*<br>#	Yes<br>Yes	-
3g	TH+ fibers V striatum	Two-way ANOVA	Interaction effect: 0.0399	*	Yes	ns
4a	Total distance travelled (Open Field)	Two-way ANOVA	Genotype effect <0.0001<br>Treatment effect:0.0006	****<br>###	Yes<br>Yes	-
4b	Total number of rearings (Open Field)	Two-way ANOVA	Interaction effect: 0.0426<br>Genotype effect<0.0001	*<br>####	Yes<br>Yes	WT CTL vs BAC CORT: p=0.0003 (***)<br>WT CORT vs BAC CORT: p=0.0001 (***)<br>BAC CTL vs BAC CORT: p=0.0404 (*)
4c	Postural instability	Two-way ANOVA	Treatment effect: 0.0215	#	Yes	-
4d	Gait analysis: Stride length (Right Forelimb)	Two-way ANOVA	Interaction effect: 0.0494<br>Genotype effect: 0.0064<br>Treatment effect:0.04	*<br>##<br>*	Yes<br>Yes<br>Yes	WT CTL vs WT CORT: P=0.0558 (ns)<br>WT CTL vs BAC CTL: P=0.0199 (*)<br>WT CTL vs BAC CORT: P=0.0071 (**)
4d	Gait analysis: Stride length (Left Forelimb)	Two-way ANOVA	Genotype effect: 0.0047	**	Yes	-
4d	Gait analysis: Stride length (Right Backlimb)	Two-way ANOVA	Genotype effect: 0.0083<br>Treatment effect:0.378	**<br>#	Yes<br>Yes	-
4d	Gait analysis: Stride length (Right Backlimb)	Two-way ANOVA	Genotype effect: 0.007	**	Yes	-
5a	CRF expression in rat hippocampus	Unpaired t-test	0.0049	**	Yes	Does not apply
5c	MR expression in rat hippocampus	Unpaired t-test	0.0755	ns	No	Does not apply
5d	CRF expression in human hippocampus	Unpaired t-test	0.0424	*	Yes	Does not apply
5e	GR expression in human hippocampus	Unpaired t-test	0.0512	ns	No	Does not apply
5f	MR expression in human hippocampus	Unpaired t-test	0.0408	*	Yes	Does not apply
5g	Human asyn expression in human hippocampus	Unpaired t-test	0.0436	*	Yes	Does not apply
S2	Elevated Plus Maze (total distance travelled)	Two-way ANOVA	Genotype effect: 0.0008<br>Treatment effect: 0.0127	***<br>#	Yes<br>Yes	-
S4	Noradrenaline (NA) in the hippocampus	Two-way ANOVA	Genotype effect: 0.0064	**	Yes	-
S5i	Total asyn in the hippocampus (ratio)	Two-way ANOVA	Genotype effect: 0.0939	ns	No	-
S5j	pS129 asyn in the hippocampus (ratio)	Unpaired t-test	Genotype effect: 0.0505	ns	No	Does not apply
S5k	Total asyn in the striatum (ratio)	Two-way ANOVA	Interaction effect: 0.0490<br>Genotype effect: 0.0204	*<br>*	Yes<br>Yes	WT CTL vs BAC CTL: P=0.0416 (*)
S6b	Astrocytes (GFAP) expression (in the SNpc)	Two-way ANOVA	Treatment effect:0.0327	#	Yes	-
S6e	GFAP in the striatum	Two-way ANOVA	Interaction effect: 0.0449	*	Yes	ns
S6g	Astrocytes (GFAP) expression (in the striatum)	Two-way ANOVA	Interaction effect: 0.0039<br>Genotype effect: 0.01	**<br>*	Yes<br>Yes	WT CTL vs WT CORT p=0.0489 (*)<br>WT CTL vs BAC CTL p=0.004 (**)<br>WT CTL vs BAC CORT p=0.0547 (ns)
S6h	Microglia (Iba-1) expression (in the striatum)	Two-way ANOVA	Interaction effect: 0.0086	**	Yes	WT CTL vs WT CORT p=0.0809 (ns)
