# Supplementary material for: Alpha-synuclein-induced stress sensitivity renders the Parkinson’s disease brain susceptible to neurodegeneration
Source: Acta Neuropathol Commun. 2024 Jun 17;12:100. doi: 10.1186/s40478-024-01797-w (PMC11181569; doi:10.1186/s40478-024-01797-w)
Supplement: Supplementary file 6 — Additional file 6: Figure S2. Total distance travelled (cm) in the EPM by WT and BAC animals after two weeks of CORT administration. Two-way ANOVA was applied with Bonferroni’s multiple comparisons post-hoc tests. Asterisk (*) is used to mark genotype effects while hashtag (#) marks treatment main effects. All data are expressed as Mean ± SEM. Significance levels: # p < 0.05; *** p < 0.001. N = 7–8. [file 40478_2024_1797_MOESM6_ESM.pdf]

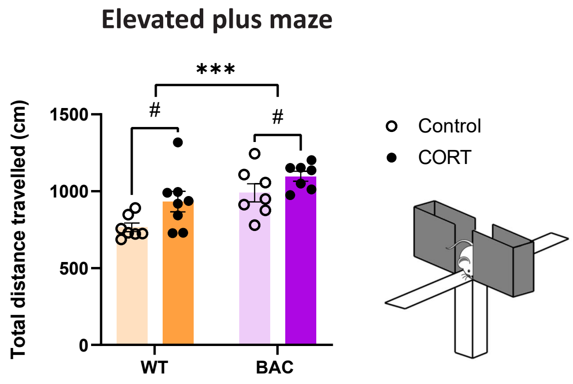

Additional file 6: Figure S2. Total distance travelled (cm) in the EPM by WT and BAC animals after two weeks of CORT administration. Two-way ANOVA was applied with Bonferroni's multiple comparisons post-hoc tests. Asterisk (*) is used to mark genotype effects while hashtag (#) marks treatment main effects. All data are expressed as Mean ± SEM. Significance levels: # $p<0.05$; *** $p<0.001$. N=7-8.
