# Supplementary material for: Alpha-synuclein-induced stress sensitivity renders the Parkinson’s disease brain susceptible to neurodegeneration
Source: Acta Neuropathol Commun. 2024 Jun 17;12:100. doi: 10.1186/s40478-024-01797-w (PMC11181569; doi:10.1186/s40478-024-01797-w)
Supplement: Supplementary file 7 — Additional file 7: Figure S3. Simple linear regression correlation analysis of the time (s) spent in the center of the OF arena to the total distance travelled (cm) in the OF arena for the animals of all the experimental groups, regardless of genotype and treatment group. Linear regression analysis was applied. N = 26. [file 40478_2024_1797_MOESM7_ESM.pdf]

## Correlation of the time spent in center to the total distance travelled in the open field arena

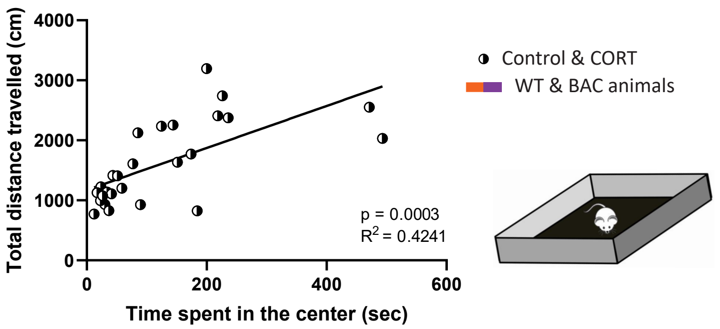


**Additional file 7: Figure S3.** Simple linear regression correlation analysis of the time (s) spent in the center of the OF arena to the total distance travelled (cm) in the OF arena for the animals of all the experimental groups, regardless of genotype and treatment group. Linear regression analysis was applied. N=26
