# Supplementary material for: Alpha-synuclein-induced stress sensitivity renders the Parkinson’s disease brain susceptible to neurodegeneration
Source: Acta Neuropathol Commun. 2024 Jun 17;12:100. doi: 10.1186/s40478-024-01797-w (PMC11181569; doi:10.1186/s40478-024-01797-w)
Supplement: Supplementary file 8 — Additional file 8: Figure S4. NA levels (mg/g wet tissue) in the hippocampus of WT and BAC animals after two weeks of CORT administration. Two-way ANOVA was applied with Bonferroni’s multiple comparisons post-hoc tests. All data are expressed as Mean ± SEM. Asterisk (*) is used to mark genotype effects. Significance levels: **p < 0.01. N = 3–4. [file 40478_2024_1797_MOESM8_ESM.pdf]

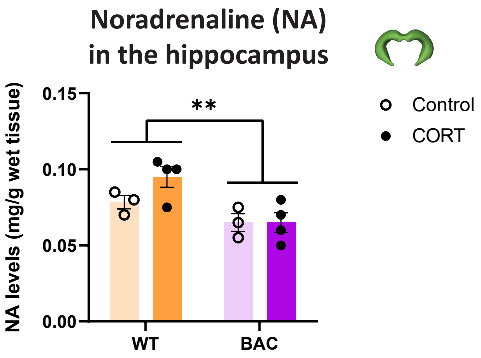

Additional file 8: Figure S4. NA levels (mg/g wet tissue) in the hippocampus of WT and BAC animals after two weeks of CORT administration. Two-way ANOVA was applied with Bonferroni's multiple comparisons post-hoc tests. All data are expressed as Mean ± SEM. Asterisk (*) is used to mark genotype effects. Significance levels: **$p<0.01$. N=3-4.
