# Supplementary material for: Alpha-synuclein-induced stress sensitivity renders the Parkinson’s disease brain susceptible to neurodegeneration
Source: Acta Neuropathol Commun. 2024 Jun 17;12:100. doi: 10.1186/s40478-024-01797-w (PMC11181569; doi:10.1186/s40478-024-01797-w)
Supplement: Supplementary file 9 — Additional file 9: Figure S5. By immunoblot biochemical analysis we measured relative protein levels (vs b-actin) of a total asyn levels in the soluble fraction of hippocampus, b pS129 asyn levels in the soluble fraction of hippocampus, c total asyn levels in the soluble fraction of striatum, d pS129 asyn levels in the soluble fraction of striatum, e total asyn levels in the insoluble fraction of hippocampus, f pS129 asyn levels in the insoluble fraction of hippocampus g total asyn levels in the insoluble fraction of striatum and h pS129 asyn levels in the insoluble fraction of striatum. We calculated insoluble to soluble fraction ratios of i total asyn levels in hippocampus, j pS129 asyn levels in hippocampus, k total asyn levels in striatum and l pS129 asyn levels in striatum. The relative quantification of protein levels was performed with the use of Fiji/ImageJ. Two-way ANOVAs were applied for (a), (c), (e), (g), (i) and (k) with Bonferroni’s multiple comparisons post-hoc tests while unpaired t-tests were applied for the comparisons (b), (d), (f), (h), (j) and (l). Asterisk (*) is used to mark genotype effects. All data are expressed as Mean ± SEM. Significance levels: * p < 0.05; ** p < 0.01; *** p < 0.001. N = 3-5. [file 40478_2024_1797_MOESM9_ESM.pdf]

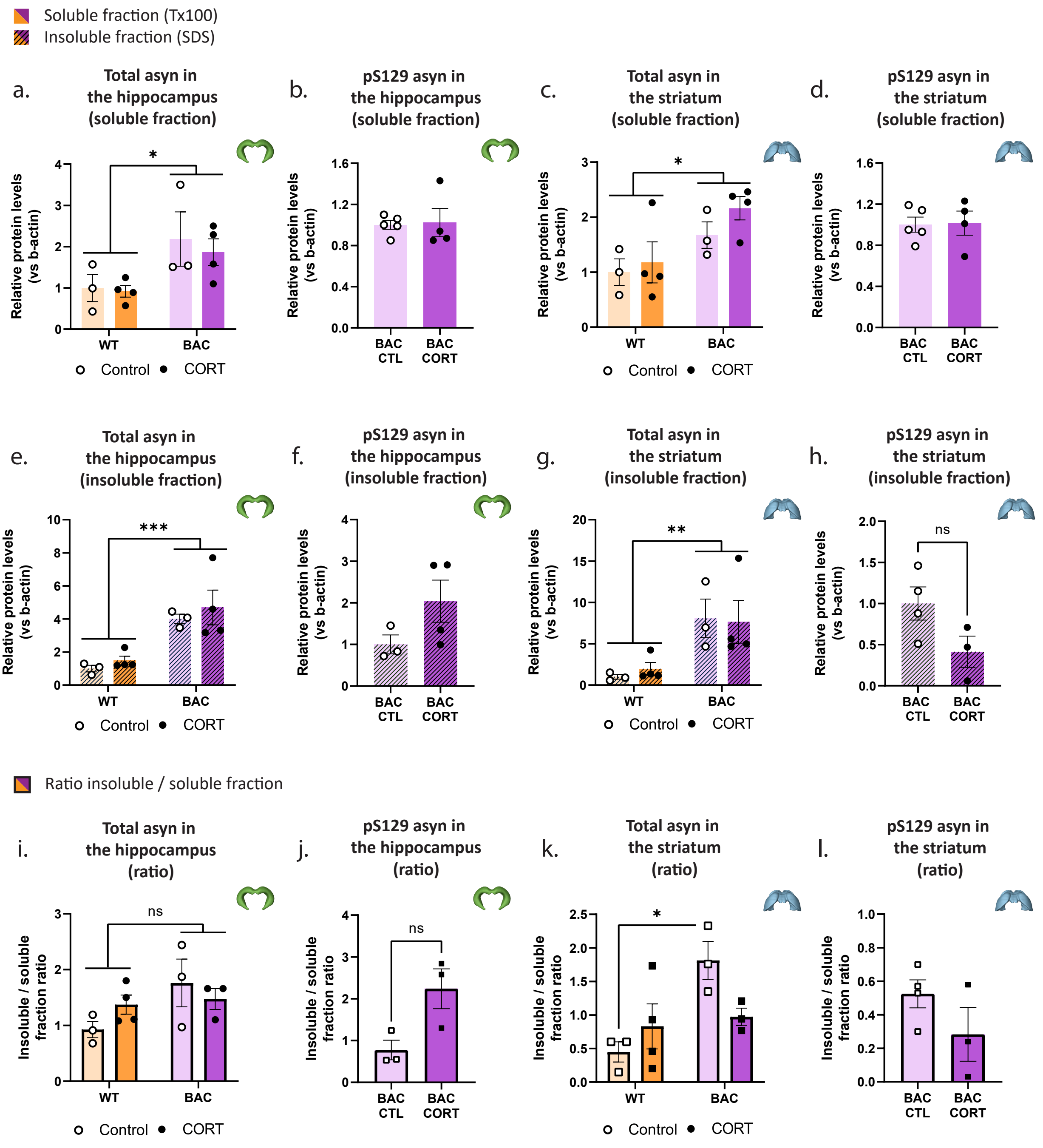

Additional file 9: Figure S5. By immunoblot biochemical analysis we measured relative protein levels (vs b-actin) of (a) total asyn levels in the soluble fraction of hippocampus, (b) pS129 asyn levels in the soluble fraction of hippocampus, (c) total asyn levels in the soluble fraction of striatum, (d) pS129 asyn levels in the soluble fraction of striatum, (e) total asyn levels in the insoluble fraction of hippocampus, (f) pS129 asyn levels in the insoluble fraction of hippocampus (g) total asyn levels in the insoluble fraction of striatum and (h) pS129 asyn levels in the insoluble fraction of striatum. We calculated insoluble to soluble fraction ratios of (i) total asyn levels in hippocampus, (j) pS129 asyn levels in hippocampus, (k) total asyn levels in striatum and (l) pS129 asyn levels in striatum. The relative quantification of protein levels was performed with the use of Fiji/ImageJ. Two-way ANOVAs were applied for (a), (c), (e), (g), (i) and (k) with Bonferroni's multiple comparisons post-hoc tests while unpaired t-tests were applied for the comparisons (b), (d), (f), (h), (j) and (l). Asterisk (*) is used to mark genotype effects. All data are expressed as Mean ± SEM. Significance levels: * $p<0.05$; ** $p<0.01$; *** $p<0.001$. N=3-5
